# CIP/KIP and INK4 families as hostages of oncogenic signaling

**DOI:** 10.1186/s13008-024-00115-z

**Published:** 2024-04-01

**Authors:** Lucia Csergeová, David Krbušek, Radoslav Janoštiak

**Affiliations:** BIOCEV—First Faculty of Medicine, Charles University, Prague, Czechia

**Keywords:** Cyclin-dependent kinase inhibitors, CIP/KIP, INK4, Oncogenic signaling, Posttranslational modification, Cancer, Therapy

## Abstract

CIP/KIP and INK4 families of Cyclin-dependent kinase inhibitors (CKIs) are well-established cell cycle regulatory proteins whose canonical function is binding to Cyclin-CDK complexes and altering their function. Initial experiments showed that these proteins negatively regulate cell cycle progression and thus are tumor suppressors in the context of molecular oncology. However, expanded research into the functions of these proteins showed that most of them have non-canonical functions, both cell cycle-dependent and independent, and can even act as tumor enhancers depending on their posttranslational modifications, subcellular localization, and cell state context. This review aims to provide an overview of canonical as well as non-canonical functions of CIP/KIP and INK4 families of CKIs, discuss the potential avenues to promote their tumor suppressor functions instead of tumor enhancing ones, and how they could be utilized to design improved treatment regimens for cancer patients.

## Background

**Fig. 1:**
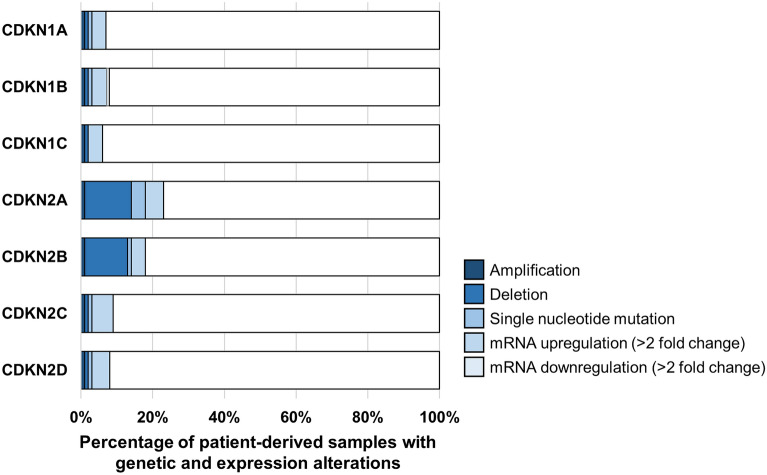
Frequency of genetic alteration and expression changes of CKIs across 32 types of cancer. Frequencies of gene amplification, homo deletion, single nucleotide mutations and mRNA expression were extracted from TCGA database containing 10,967 patient-derived samples. The frequencies are represented as 100% stacked bar chart

Cyclin-dependent kinase inhibitors are small nucleocytoplasmic proteins belonging to two families–CIP/KIP family encompassing p21CIP, p27KIP1 and p57KIP2, and INK4 family encompassing p16INK4a, p15INK4b, p18INK4c and p19INK4d. CIP/KIP family members are able to bind all the major cell cycle promoting Cyclin/CDK complexes (Cyclin A, B, D, E with respective CDK1,2,4,6), and depending on their posttranslational modification, they can either inhibit or promote the activity of bound Cyclin/CDK complexes. On the other hand, INK4 family members can bind only to Cyclin D/CDK4/6 complexes and work as inhibitors. In non-transformed cells, members of both families usually collaborate to regulate development, differentiation, and stem cell pool and have partially overlapping functions. However, in transformed cells, almost all the members are gaining new tumor-enhancing functions such as increasing survival, stimulating DNA repair, and apoptosis resistance. Since cancer cells usually retain the functionality of at least some CKI members which is evident from the low frequency of gene deletion across different cancer types (Fig. 1), it likely represents an adaptive trait increasing survival, especially in stressed conditions. Therefore, there could be a therapeutic opportunity to shift the balance from the tumor-enhancing function of these proteins to the tumor-suppressor functions, which will be discussed throughout this review.

### CIP/KIP family

**Fig. 2:**
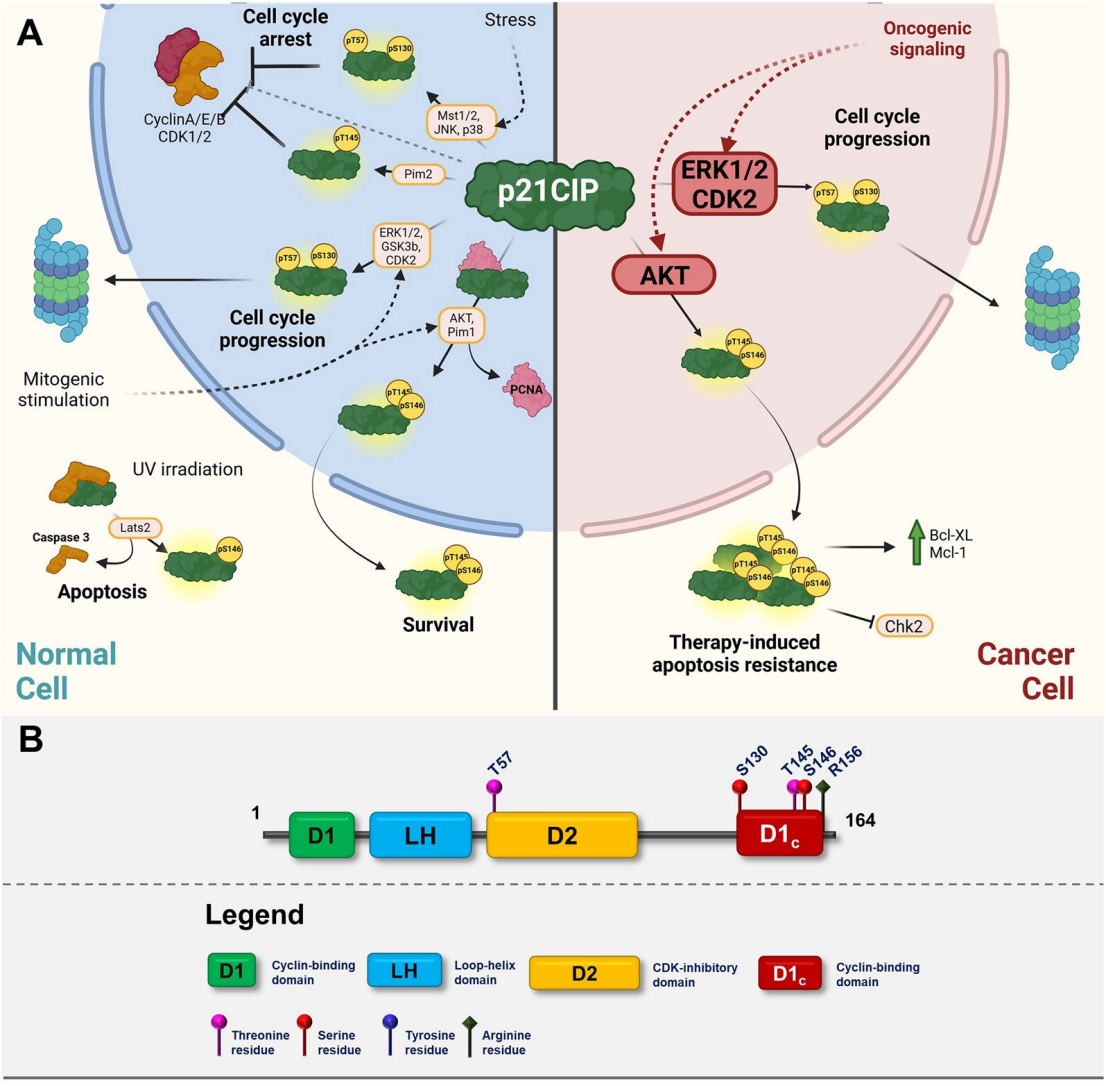
**A** Graphic illustrating the role of p21CIP in normal cells (left cellular part) and in transformed cells (right cellular part). The graphics focuses on major posttranslational modifications altering the function of p21CIP and depicting major oncogenic pathways responsible for inactivation or gain-of-function modifications. Created with BioRender.com. **B** p21CIP domain structure with highlighted sites of posttranslational modifications

**Fig. 3:**
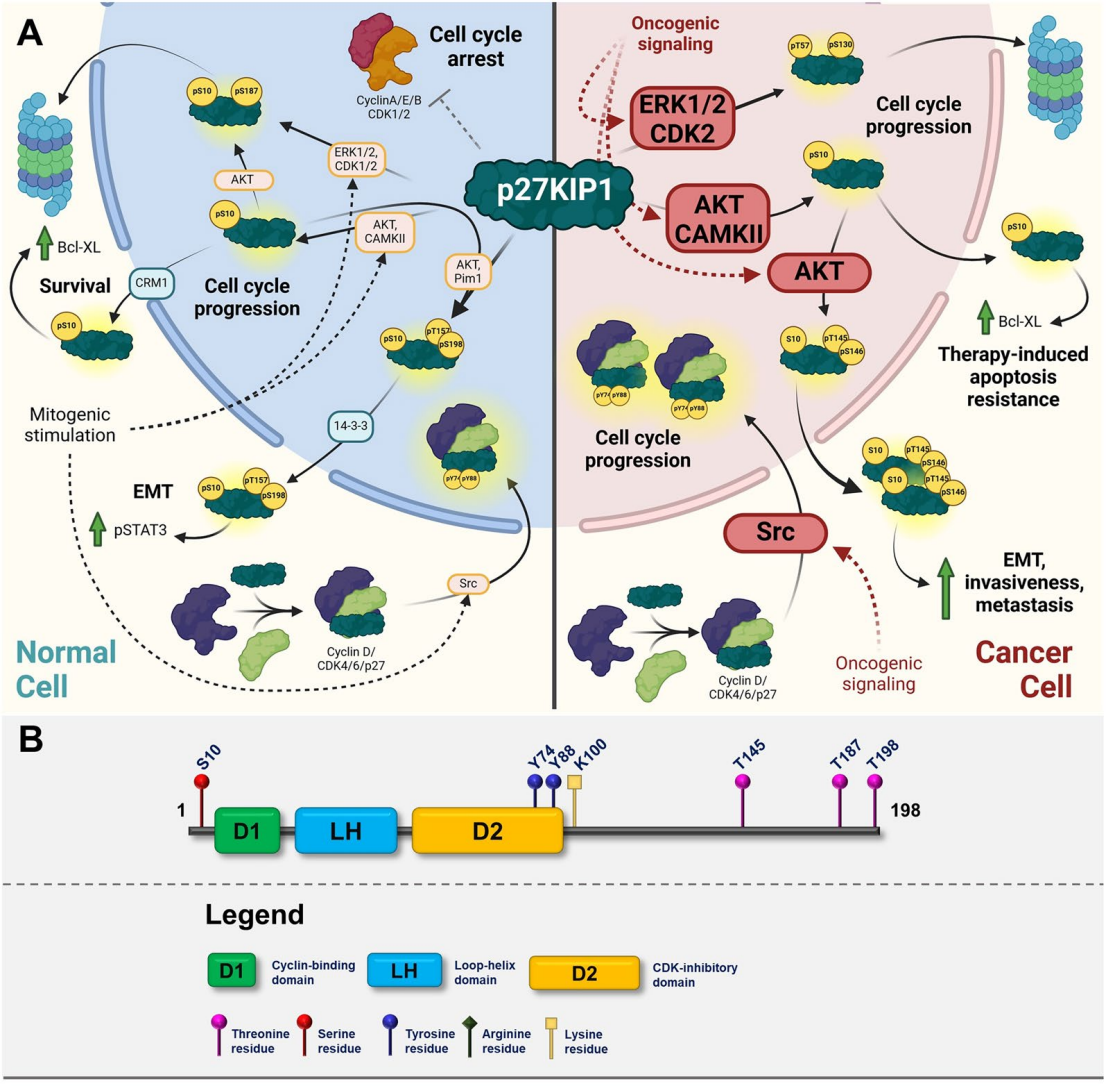
**A** Graphic illustrating the role of p27KIP1 in normal cells (left cellular part) and in transformed cells (right cellular part). The graphics focuses on major posttranslational modifications altering the function of p27KIP1 and depicting major oncogenic pathways responsible for inactivation or gain-of-function modifications. Created with BioRender.com. **B** p27KIP1 domain structure with highlighted sites of posttranslational modifications

**Fig. 4:**
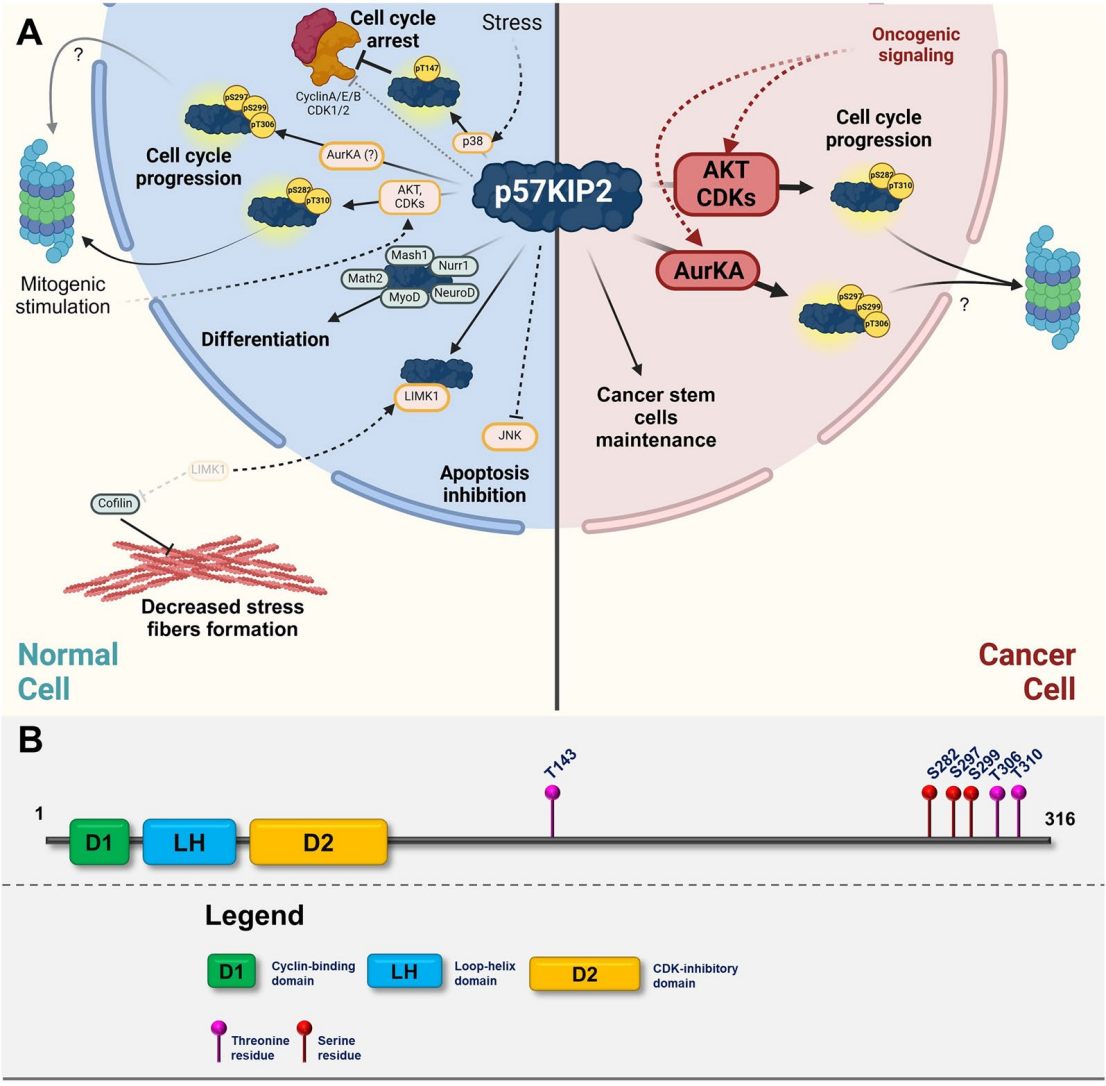
**A** Graphic illustrating the role of p57KIP2 in normal cells (left cellular part) and in transformed cells (right cellular part). The graphics focuses on major posttranslational modifications altering the function of p57KIP2 and depicting major oncogenic pathways responsible for inactivation or gain-of-function modifications. Created with BioRender.com. **B** p57KIP2 domain structure with highlighted sites of posttranslational modifications

The canonical function of CIP/KIP family proteins–p21CIP, p27KIP1 and p57KIP2 is regulation of cell cycle progression through binding different complexes of Cyclins and CDKs [1–3]. Proteins of this family have been mostly studied as inhibitors of CDK complexes; however, they can also act as positive regulators through stabilizing heterodimers of Cyclins and CDKs [4, 5]. The CIP/KIP proteins share a high level of structural similarity in their N-terminal domains that mediates their CDK-inhibitory actions (kinase inhibitory domains–KIDs) [6, 7]. On the other hand, they contain intrinsically disordered C-terminal domain (CTD), which differs in sequence among individual members and has an important regulatory role [8, 9]. Members of this family have slightly different roles in normal development, which is illustrated by different developmental abnormalities in mice lacking individual members of the CIP/KIP family [10]. Moreover, these proteins are associated with CDK complexes throughout the whole cell cycle and exert their inhibitory activities only under specific conditions that lead to increased concentration and differential posttranslational modifications [6] (Figs. 2, 3, 4).

### p21CIP

#### Function

The canonical function of p21CIP in normal, not transformed cells is to regulate the cell cycle mainly through interaction with Cyclin-CDK complexes. P21CIP accumulates in normal cells arrested in G0 phase and inhibits the entry into cell cycle through inhibition of CDK4/6 complexes [11]. In response to environmental stress it binds also to CDK1 complexes and arrest cells in G2 phase [12]. P21CIP binds to most of the major Cyclin/CDK complexes (Cyclin D/CDK4/6, Cyclin E/CDK2, Cyclin A/CDK2) [13] and the binding of p21CIP to Cyclin/CDK complexes is mediated through Cyclin-binding and CDK-binding motifs. P21CIP contains two Cyclin binding motifs–N-terminally located Cy1 is the major interaction site, and C-terminal Cy2 is an additional binding site that provides further refinement of binding [14]. Optimal inhibition is further ensured by binding the KID domain to CDK [15]. Additionally, p21CIP inhibits Cyclin/CDK complexes indirectly, either through displacing cdc25A necessary for full activation of Cyclin E/CDK2 [16] or through inhibition of CDK-activating kinase (CAK), which is crucial for phosphorylation of Thr161 on CDK1 and its full activation [17]. On the other side, p21CIP induces assembly and activation of Cyclin D/CDK4 complexes, which can sequester p21CIP from Cyclin/CDK2 complexes, leading to their increased activity and progression through the cell cycle [7, 18]. However, the mechanism of p21CIP-mediated activation of Cyclin D/CDK4/6 is not fully elucidated but likely would be mediated by specific p21CIP phosphorylation similar to p27KIP1 [19]. p21CIP also regulates DNA damage repair, mainly through interaction with PCNA. P21CIP sequesters PCNA and inhibits the formation of complexes of PCNA with DNA repair machinery that leads to impairment of mismatch repair [20]. p21CIP accumulates also on the sites of double-strand breaks and promotes homologous recombination (HR) or non-homologous end joining (NHEJ) depending on the cell cycle phase [21, 22]. The role of p21CIP in response do DNA damage is further complicated because p21CIP can either repress or promote apoptosis due to DNA damage [14].

It is apparent that the role of p21CIP in regulation of both CDK activation or inhibition as well as in DNA damage repair and response is dependent on the actual cellular state and dominant signaling, and is regulated by p21CIP phosphorylation and localization.

#### Expression and stability regulation

p21CIP is encoded by *CDKN1A* gene that is located at chromosome 6p21.2 in humans and the key transcription factor regulating p21CIP expression is p53 [23]. In the canonical scenario, p53 is stabilized and activated in response to various extrinsic (chemicals, radiation) and intrinsic (replication stress, DNA damage) stress signals, which leads to increased expression of p21CIP. Increased abundance of p21CIP inhibits Cyclin/CDK complexes (CDK1, CDK2, CDK4/6) and promotes cell cycle arrest in G1, S, and G2 phases [24]. Therefore, numerous proteins that stimulate p53 stability and transcriptional activity, such as ATM, BRCA1, GADD34, or KLF4, also regulate p21CIP abundance [24]. In addition to p53-dependent transcription, several p53-independent mechanisms stimulate p21CIP expression. Major transcription factors that regulates p21CIP expression, in the absence as well as presence of functional p53, are Sp1 and Sp3 [25]. Sp1 is activated in response to oncogenic signaling as a fail-safe response that blocks the carcinogenesis through p21CIP activation [26]. Similarly, Sp1 collaborates with TGFβ signaling and activates p21CIP expression to block the cell cycle progression [27, 28]. Lastly, the major tumor suppressor protein–Rb1–is also collaborating with Sp1 to induce p21CIP expression thus inhibiting the cell cycle progression [28–31] Pathways regulating p21CIP expression are upregulated in response to various environmental stresses as well as oncogenic pathway overactivation the expression of p21CIP is a major event leading to cell cycle arrest in the response to stress or oncogene activation. However, the fact that p21CIP is widely expressed in human cancers indicates that posttranslational regulation is the key determinant of p21CIP function.

On the post-translationally level p21CIP is mainly regulated by three major ubiquitin complexes through the cell cycle: SCF^Skp2^-Cks1, Cul4-DDB1^Cdt2^, and APC/C^Cdc20^. Cul4^Cdt2^ and SCF^Skp2^ ubiquitin ligase degrade p21CIP, mainly in the S-phases of the cell cycle. Cul4-mediated degradation of p21CIP is triggered by PCNA, which ensures transition through S-phase in the absence of stress signals (e.g., DNA damage) [32, 33]. Similarly, the SCF complex destabilizes p21CIP throughout G1 and S-phase, which leads to cell cycle progression [34–36]. The last major ubiquitin ligase complex, APC/C^Cdc20^, controls the ubiquitin-mediated degradation of p21CIP in prometaphase [37]. Little is known about deubiquitination of p21CIP. So far the only identified p21CIP-specific deuibiquitinase is USP11. USP11 is activated in response to DNA damage and deubiquitinates p21CIP, which leads to its stabilization and facilitation of cell cycle arrest and DNA damage repair [38]. Activity of p21CIP-destabilizing systems is increased in cancer cells which is one of the avenues to bypass upregulation of p21CIP transcription and promote cancer growth. Moreover, the degradation of p21CIP is further stimulated through its posttranslational modifications, which will be discussed in the following sections.

#### Posttranslational modifications

Despite p21CIP being a relatively small protein, it is highly modified post-translationally, mostly through phosphorylation. Multiple signaling pathways target p21CIP, which serves as a hub for integrating different signals to regulate the cell cycle, DNA damage repair, transcription, or apoptosis. There are four major sites of phosphorylation on p21CIP–T57, S130, and T145/S146 (Fig. 2A, B). Threonine 57 is targeted by GSK3β and ERK1/2, which leads to its export to the cytoplasm, destabilization, and degradation [39, 40]. On the other side, phosphorylation of the same Thr57 by Mst1/2, JNK, or p38 leads to its stabilization and cell cycle arrest [41, 42]. The same context dependency has been described also for other phosphorylation sites. Phosphorylation of Ser130 by ERK1/2 or Cyclin E/CDK2 leads to p21CIP degradation, and the same phosphorylation mediated by JNK and p38 stabilizes p21CIP [42–44]. Similarly, phosphorylation of Thr-145 and Ser146 by different kinases has different effect. The major kinase promoting phosphorylation of these residues is Akt, and phosphorylation of Thr145 and Ser146 by Akt leads to disruption of binding to PCNA, p21CIP stabilization, and export to the cytoplasm where it exerts its pro-survival functions [45, 46]. Since Akt is commonly upregulated in various cancers, phosphorylation of p21CIP on Thr145 and Ser146 is clear demonstration how oncogenic signaling not only inhibits anti-proliferative functions of p21CIP, but also stimulates its pro-survival role. Another kinase that was shown to promote phosphorylation of these sites is Pim1. In vivo, Pim1 stimulates phosphorylation of both sites however, in vitro experiments show that Pim1 only phosphorylates Thr145, which leads to p21CIP stabilization [47]. This seems to promote S146 phosphorylation and export to cytoplasm and stimulation of survival in response to stress [47]. On the other hand, phosphorylation of Thr145 in the context of active Pim2 leads to stabilization of p21CIP and cell cycle arrest [48]. Interestingly, this phosphorylation is not associated with the phosphorylation of S146 nor with p21CIP export to the cytoplasm. Finally, Ser146 is also phosphorylated by Lats2 after UV irradiation independently of Thr145 phosphorylation, and it leads to its decreased stability [49]. This decreased stability is translated into a higher rate of apoptosis, which is likely caused by decreased p21CIP-mediated caspase inhibition [49]. The importance of the Thr145/Ser146 phosphorylation is also substantiated by the fact that Thr145 phosphorylation is promoted by methylation of Arg156 by PRMT6, which leads to p21CIP cytoplasmic localization and increased survival [50]. In summary, the output of p21CIP phosphorylation depends on multiple factors, such as the interaction between different phosphorylation sites, cellular context and type, and the trigger (Fig. 2A). Therefore posttranslational modification of p21CIP, mainly phosphorylation, could overcome increased expression that is stimulated by various stresses or even the overactivation of the common oncogenes.

#### Role in cancer

Since the p21CIP function is highly context-dependent, it is no surprise that the role of p21CIP in cancer is not as straightforward as initially thought. In mouse models of carcinogenesis, deletion of p21CIP led to higher susceptibility to development of hematologic, epithelial and endothelial tumors [51, 52], and these mice are more prone to develop colonic tumors after treatment with chemical carcinogen [53]. Interestingly, the role of p21CIP is quite different in established tumors treated with anticancer drugs. Various in vitro and in vivo models showed that tumor cells with deletion of p21CIP are more susceptible to chemotherapy such as Chk1 inhibitors, platinum-based compounds, microtubule inhibitors or irradiation [54–57]. In comparison to mice-derived results, human cancer data shows that it is more complicated [58]. TCGA database analysis shows that the CDKDN1A gene is mutated only in ~ 2% of cases across different cancer types, and more than half of those mutations are gene amplifications. Additionally, mRNA expression is associated with worse overall survival in patients with gastrointestinal tumors, lung adenocarcinoma, or breast cancer [58, 59]. As it is established that p21CIP concentration is a crucial determinant of its function, it is maintained at low levels in the majority of tumor types. For example, in osteosarcoma and lung adenocarcinoma, p21CIP is maintained at low levels by increased expression of Cul4-DDB1^CTD2^ E3 ubiquitin ligase, resulting in cell cycle progression [60, 61]. Similarly, in melanoma, the expression of p21CIP is inhibited by the epigenetic regulator EZH2, and EZH2 knockdown in melanoma cells rescues p21CIP expression [62]. In contrast to its proposed tumor suppressor role, p21CIP can promote tumorigenesis mainly when localized in the cytoplasm, which is in agreement with the evidence that major survival pathways (Akt, p38) induce phosphorylation of p21CIP and its export to cytoplasm [45]. For example, p21CIP also plays a role in oncogenesis while located in the cytoplasm, where it upregulates pathways associated with survival and apoptosis resistance [63, 64]. It is becoming clear that transient overexpression of p21CIP in certain cell populations and states and its posttranslational modification is more important for its role in cancer than steady-state levels. An increasing amount of evidence shows that p21CIP promotes drug resistance mainly through two distinct mechanisms in two different subcellular compartments. Cytoplasmic localization of p21CIP regulated by its T145 phosphorylation confer resistance to several anticancer treatments. It stimulates resistance to paclitaxel and cisplatin in ovarian cancer [65, 66] or resistance of colorectal cancer to 5-fluorouracil [67]. In cytoplasm, p21CIP can directly bind and inhibit pro-apoptotic mitochondrial pathways [68, 69] or inhibit translocation of proapoptotic proteins into the nucleus by masking their nuclear localization signal [67]. While cytoplasmic p21CIP functions are largely independent of its cell cycle inhibitory function, p21CIP can indirectly promote therapy survival of cancer cells through cell cycle arrest and increased protection of cells from DNA damage [70, 71]. Additionally, p21CIP-mediated cell cycle arrest is crucial for the maintenance of stem cells in certain conditions. In hematopoietic stem cells, p21CIP expression ensures their dormant phenotype and is crucial for maintaining the hematopoietic stem cell pool [72]. Similarly, p21CIP could function as a regulator of cancer cell stemness. However as of now, there is no clear evidence confirming or disproving this, and more research is needed.

Overall the role of p21CIP in human cancer is complex but several conclusions could be suggested. Induction of p21CIP transcription after cellular stress (DNA damage, oxidative stress, oncogene overactivation) promotes cell cycle arrest and protects organisms from tumorigenesis. However, cancer cells bypass this restriction by either maintaining low levels of p21CIP through increased degradation or modifying its function through specific phosphorylation. Moreover, specific phosphorylation of p21CIP stimulated by oncogenic signaling not only inhibits its tumor suppressor function, but also stimulates its tumor promoting role such as apoptosis protection.

### p27KIP

#### Function

Protein p27KIP1, encoded by the *CDKN1B* gene, which is located at chromosome 12, was first identified as the CDK2 inhibitor when treating mice epithelial cells with Tumor Growth Factor β (TGF- β) [73, 74]. Since then, multiple cellular functions in the regulation of growth and development have been attributed to p27KIP1 deficient mice [51, 75–77]. As it belongs to the CIP/KIP family of CDK inhibitors, its canonical function is to bind Cyclin/CDK complexes and regulate their functions [78]. Like the other members of the CIP/KIP family, it possesses a binding domain as well as a CDK inhibitory domain (KID) that contains regulatory tyrosines [79, 80].

In its active form, p27KIP1 binds and inhibits Cyclin E(A)/CDK2, Cyclin (B)/CDK1, and Cyclin D/CDK4/6 complexes [80]. However, more detailed studies have revealed that the function of p27KIP1 is modulated by its phosphorylation. In the case of Cyclin D/CDK4, unphosphorylated p27KIP1 promotes the assembly of Cyclin D/CDK4/6 complexes, and its phosphorylation on Thr157 and Thr198 by Akt greatly enhances its scaffolding activity [4, 81]. However, these complexes are only partially active, and only subsequent phosphorylation by non-receptor tyrosine kinases (NRTK) on tyrosines 74 and 88 leads to conformational change and subsequently to full activation, where the trimeric complex is more active than Cyclin D/CDK4 heterodimer itself [19, 82]. Similarly, p27KIP1 that is not phosphorylated on Tyr74 and Tyr88 binds to Cyclin E/A/B–CDK1/2 complexes and inactivates them. Subsequent phosphorylation of those tyrosine residues leads to trimeric complex dissociation, stimulation of threonine 187 phosphorylation by liberated Cyclin E/A/B–CDK1/2 complexes and p27KIP1 degradation which leads to cell cycle progression [79, 80]. Therefore, the key function of p27KIP1 is to regulate the cell cycle progression, especially the exit from the cell cycle into quiescence and maintaining the G0 phase. This role is the most dominant in non-transformed cells, however, p27KIP1 is also important for cancer cell quiescence regulation in conjunction with adjacent pathways [78]. Beyond inhibition of cell cycle progression, several other nuclear and cytoplasmic functions of p27KIP1 were identified. In the nucleus, p27KIP1 engages in the regulation of gene transcription both as a co-repressor and as a co-activator. P27KIP1 associates with p130, E2F4, HDAC1, and Sin3A in quiescent cells and regulates gene expression leading to a more profound G0 phase [83]. Moreover, there is an interplay between p27KIP1 and p300/CBP-associated factor (PCAF) at PCAF-regulated gene promoters where p27KIP1 represses the transcription, and PCAF activates it, which serves as a fine-tuning of target gene expression [84]. On the other hand, p27KIP1 emerged as an important regulator of EMT through the co-activation of c-Jun-mediated transcription programs. C-terminally phosphorylated p27KIP1 (p27pT157pT198) is recruited to chromatin together with c-Jun and upregulates TGF-β2 expression leading to TGFβ signaling activation, EMT, and invasion [85]. Similarly, cytoplasmic c-terminally phosphorylated p27KIP1 promotes EMT through STAT3. P27pTpT interacts with JAK2, which leads to increased phosphorylation of STAT3, its translocation to the nucleus, and activation of expression of TWIST, which subsequently promotes EMT and invasiveness [86]. p27KIP1 also promotes the turnover of actin and actin cytoskeleton through RhoA regulation. P27KIP1 binds directly to RhoA and inhibits its association with Rho-GEFs (guanine nucleotide exchange factors), leading to downregulation of ROCK1 activity and increased actin cytoskeleton turnover [87]. Interestingly, similar to the cytoplasmic function of p21CIP, serine 10 phosphorylated p27KIP1 has been shown to be localized to cytoplasm and promote apoptosis resistance of HeLa cells [88]. Finally, p27KIP1 has been implicated in the control of autophagy and vesicular trafficking. P27KIP1 binds to LAMTOR1, a scaffolding protein important for RAGULATOR complex and downstream mTORC1 activation. P27KIP1 binding to LAMTOR1 inhibits its GAP activity (GTPase activation protein), thus prolonging RHEB GTP loading and subsequent mTORC1 activity [89, 90]. To conclude, even though p27KIP1 default function is inhibition of Cyclin/CDK complex activity, this function is greatly influenced by its posttranslational modification. These modifications are mostly prominent in transformed cells where overactivated oncogenic kinases such as Src or Akt are switching off its tumor suppressor role and turning on its tumor promoting role.

#### Expression and stability regulation

Similar to p21CIP, the expression and stability of p27KIP1 are tightly regulated mostly by mitogenic signaling. Mitogen-activated signaling pathways, such as MAPK or PI3K/Akt, inhibit the transcription of p27KIP1 mRNA through the action of several transcriptional repressors. One of the major repressors inhibiting p27KIP1 transcription is AP-1 TF consisting of c-Jun and c-Fos, which binds the *CDKN1B* promoter after mitogenic stimulation and blocks p27KIP1 mRNA transcription [91]. In contrast, expression of Forkhead transcription factors (AFX, FKHR, and FKHR-L1) stimulates the expression of p27KIP1 mRNA and blocks the transition of cell cycle in G1 phase. These factors are phosphorylated by oncogenic Akt, which stimulates their nuclear export and prevents activation of p27KIP1 mRNA expression [92]. FoxO1a, FoxO3a and FoxO4 transcription factors work in a similar fashion–they promote expression of p27KIP. There are several kinases that phosphorylates these transcription factors and prevent increased expression of p27KIP1. Two main pro-survival kinases (Akt, SGK) are phosphorylating FoxO factors which leads to their sequestration in cytoplasm and preventing stimulation of p27Kip expression. This leads to promotion of cell survival in the presence of apoptotic stimuli [93, 94]. Additionally Pim kinase family members (Pim1, Pim2, and Pim3) also phosphorylate FoxO1 and FoxO3a transcription factors and prevent p27KIP1 transcription [95]. Finally, p27KIP1 mRNA expression is also stimulated by the major pro-proliferative transcription factor E2F1, which binds to the p27KIP1 promoter and activates mRNA and protein expression of p27KIP1 [96]. Since p27Kip has differential role in regulation of cell cycle, this loop can both promote progression of cell cycle through stimulation of CDK4/6-Cyclin D activity as well as inhibit cell cycle progression and stimulate the apoptosis by blocking the activity of CDK1-Cyclin A/B complexes [96]. Posttranslational regulation of p27KIP1 protein stability is controlled more extensively. The major ubiquitin ligase responsible for p27KIP1 degradation is SCF(Skp2) in association with Cks1 [36, 97]. This complex’s activity peaks in the S-phase when p27KIP1 degradation is happening. This degradation is triggered by T187 phosphorylation by CDK2 [98]. The major compartment of p27KIP1 degradation is the cytoplasm, where another 2 complexes were identified to be responsible for its degradation. Pirh2 directly ubiquitinates p27KIP1 and targets it to degradation by proteasome. Pirh2 expression steadily increases from the late G1 phase, and it cooperates with SCF for p27KIP1 degradation throughout S-phase [99]. Lastly, the KPC ubiquitin ligase complex was identified to be responsible for p27KIP1 degradation on the transition between G0 and G1 phases, and this degradation takes place only in the cytoplasm [100]. Although p27KIP1 is a major regulator of cell quiescence, its functionality depends on the localization and posttranslational modifications.

#### Posttranslational modifications

Similarly, to p21CIP function, localization, and stability of p27KIP1 are extensively regulated by posttranslational modifications, mainly phosphorylation. There are 6 major phosphorylation residues that affect the function and stability of p27KIP1–S10, Y74, Y88, T157, T187, and T198 (Fig. 3A, B). Phosphorylation of S10 is the key event that stimulates nuclear export as well as stabilization of p27KIP1 [101]. S10 phosphorylated p27KIP1 is bound by CRM1, which facilitates its export to cytoplasm [102]. S10 phosphorylation significantly increases p27KIP1 stability and must be dephosphorylated to be degraded [101]. There are several kinases targeting this site. Akt is the major kinase phosphorylating S10, and this phosphorylation also stimulates the sequential phosphorylation of Thr187 [103]. Interestingly this phosphorylation is stimulated under physiologically relevant conditions such as oxidative stress which indicates that p27KIP cytoplasmic localization is important for cell survival [103]. Same serine 10 is also target for Calmodulin-dependent protein kinase II (CaMKII) and this phosphorylation leads to export to the cytoplasm and stimulation of apoptosis resistance [104]. Phosphorylation of S10 could be one of the first steps in the G0-G1 transition, as this phosphorylation mediates its export to the cytoplasm and stabilizes it [101]. Cytoplasmic p27KIP1 then binds to Cyclin D and CDK4/6 and promotes the assembly of the complex [4]. In cytoplasm, p27KIP1 is also phosphorylated on Y74 and Y88 by non-receptor tyrosine kinases such as Src family kinases or Abl. This phosphorylation leads to a conformational change and increased activity of the trimeric p27KIP1/Cyclin D/CDK4/6 complex [19]. Phosphorylation of T157 and T198 has a similar function to S10 phosphorylation–nuclear export. These two sites are phosphorylated by Akt and Pim kinases, which leads to the binding of 14–3–3 protein and export to cytoplasm [95, 105, 106]. The fate of threonine phosphorylated p27KIP1 then depends on the cellular context. Phosphorylation of T198 increases its association with Skp2, which might lead to its degradation. However, double phosphorylated p27KIP1 (pT157pT198) promotes cell motility and EMT through various mechanisms described above [86]. On the other hand, phosphorylation of T187 unambiguously leads to its Skp2-mediated degradation [107, 108]. This is in agreement with the fact that T187 is a major phosphorylation site targeted by Cyclin A(E)/CDK2 complexes [109] or ERK1/2 [110]. An additional modification that regulates p27KIP1 stability is the acetylation of lysin 100 by PCAF. P27KIP1 is acetylated mainly at the beginning of the G1 phase, leading to its proteasomal degradation, which is independent of the SCF complex [111]. Acetylated p27KIP1 could counteract stabilizing phosphorylation on T157/198 and increase its degradation at the G0-G1 transition. In summary, posttranslational modifications of p27KIP1 play crucial role in regulating its function. Overactivated pro-survival and pro-proliferative signaling pathways, seen in majority of the cancers, leads to stimulation of specific p27KIP1 phosphorylation which in turn results in cytoplasmic localization, emergence of tumor-promoting functions of p27KIP1 such as enhancing of Cyclin D/CDK4/6 complex assembly or EMT and invasiveness boosting (Fig. 3A).

#### Role in cancer

The role of p27KIP1 in cancer development is context dependent. Initial in vivo mice experiments showed that knock out of p27KIP1 leads to increased number of pituitary adenomas and are more susceptible to chemically induced colon adenocarcinomas [77, 112]. However this phenotype was not recapitulated in H-Ras stimulated lung tumors where deletion of p27KIP had no effect on number of lung tumors, in prostate mouse model of carcinogenesis (*Nkx3.1*^–/–^; *Pten*^+/–^; *p27*^–/–^) or in intestinal adenocarcinoma mouse model driven by *Smad3* deletion [51, 113, 114]. Additionally, Besson and Roberts prepared a mouse model of carcinogenesis where the wild type p27KIP1 was replaced by mutant form unable to bind canonical CDK complexes (p27^CK−^) and showed that mice with this mutation develop tumors to a much bigger extent than mice with complete p27KIP1 knockout, and these tumors are also significantly larger [53]. When looking at the human cancers, similarly to p21CIP, p27KIP1 is not extensively mutated in human cancers, indicating a context-dependent role. In agreement with its tumor-suppressing canonical function, p27KIP1 is downregulated in various cancer types, such as prostate cancer [115], lung adenocarcinoma [99, 116], colorectal carcinoma [117], bladder cancer [118], endometrial cancer [119], breast cancer [120], and glioblastoma [121], and this downregulation is associated with a worse prognosis and lower overall survival [121]. Inhibition of the tumor suppressor function of p27KIP1 in these tumors is mediated by its nuclear export and increased degradation. On the other hand, several reports show that relocation of p27KIP1 into the cytoplasm leads not just to disinhibition of nuclear CDKs, but also has pro-tumorigenic role and cytoplasmic localization of p27KIP1 is associated with poor prognosis and patient survival with osteosarcoma [122], lung carcinoma [123], hepatocellular carcinoma [124], urothelial carcinoma [86], renal carcinoma [125] and melanoma [126]. In contrast to the pro-tumorigenic role of p21CIP in promoting survival and apoptosis resistance, the pro-tumorigenic role of p27KIP1 is predominantly linked to its EMT-promoting capabilities. As discussed in the “Function” section, C-terminally double phosphorylated p27KIP1 (p27pTpT) promotes EMT and invasion through stimulation of TGF-β2 and TWIST expression [85, 86]. Indeed, high cytoplasmic expression of p27KIP1 correlates with higher metastatic spread of osteosarcoma, breast cancer, or melanoma [127–129].

Evidence to date shows that p27KIP1 has both tumor promoting as well as tumor suppressing function which depends mainly on the developmental stage of the cancer as well as on the signaling context. In the initial phases of tumor development acts as a barrier for transformation, however in later stages, it is posttranslationally modified and functions mainly to promote tumor growth and dissemination.

### P57KIP2

The third member of the CIP/KIP family, p57KIP2, is the least studied. Although it possesses non-canonical functions similar to other family members, to date, it has been described as a *bona fide* tumor suppressor. P57KIP2 is encoded by the *CDKN1C* gene located at chromosome 11 [130], and its key role is the inhibition of Cyclin/CDK complexes, mainly at the G1-S phase transition [130]. P57KIP2 interacts with Cyclins and CDK through its N-terminal domain but also contains additional domains shared with other family members and unique domains that mediate p57KIP2-specific interactions and functions that will be described later [131]. The key function of p57KIP2 is the cell cycle inhibition through CDK complexes inhibition, which is supported by the fact that forced overexpression of p57KIP2 leads to cell cycle arrest in G1 [130]. Similarly to other family members, p57KIP2 promotes the assembly of Cyclin D/CDK4/6, and it contains conserved tyrosine that could be phosphorylated and increase the activity of CDK4/6. However, there is no evidence for it yet. Additionally, p57KIP2 inhibits the cell cycle through interaction with PCNA [132]. P57KIP2 contains PCNA-binding site on its C-terminus and disruption of this binding site reduce the ability of p57KIP2 to inhibit myc and Ras-mediated transformation [132]. Regulation of the cell quiescence, stemness, and differentiation is directly linked to inhibition of the cell cycle, and p57KIP2 is implicated in the control of all of those aspects, which is further supported by the “at birth” lethality of p57KIP2 KO mice [133, 134]. p57KIP2 is mostly involved in regulating the development of central and peripheral nerve systems, musculoskeletal systems, and maintenance of the adult stem cell population [131].

Besides its role in cell cycle regulation, p57KIP2 is engaged in the regulation of other cellular processes, such as cell motility, apoptosis, or transcription. P57KIP2 contains a unique central PAPA domain that binds LIMK1 kinase [135]. LIMK1 kinase is one of the key kinases regulating the actin cytoskeleton through cofilin phosphorylation. Activation of LIMK1 leads to inactivation of cofilin and decreased actin turnover [136]. Two contradictory scenarios where p57KIP2 interacted with LIMK1 were described. Yokoo and colleagues showed that overexpression of p57KIP2 sequestered LIMK1 kinase in the nucleus, which led to decreased stress fiber formation [135]. On the contrary, Vlachos and Joseph showed that overexpression of p57KIP2 led to stimulation of LIMK1 activity in the cytoplasm without nuclear localization, which ultimately led to increased stress fiber formation and decreased cell motility [137]. These contradictory results could be explained by the fact that the first study looked at the non-transformed cells (COS-7) and the second one on transformed HeLa cells, which suggest additional regulation of p57KIP2 function in cancer cells. Supporting evidence for p57KIP2 as a motility-restricting protein came from Sakai and colleagues, who expressed p57KIP2 in glioblastoma cells and showed that it greatly decreased invasion [138]. p57KIP2 has been shown to be involved in apoptosis regulation both as positive and negative regulator. P57KIP2 is phosphorylated by p38, which stimulates its affinity for CDK2, leading to cell cycle arrest and activation of reparatory mechanisms [139]. Moreover, p57KIP2 interacts with JNK and inhibits its pro-apoptotic functions in response to UV [140]. On the other hand, few reports show that p57KIP2 overexpression potentiates the effect of genotoxic stress, however the mechanistic elucidation of the signaling is not compelling [141, 142]. Like p27KIP1, p57KIP2 can directly bind several transcription factors and regulate their function, mostly associated with development and differentiation. P57KIP2 binds MyoD, which results in its stabilization and increased MyoD-mediated transcription, which leads to myogenic differentiation [143]. p57KIP2 is crucial for neuronal differentiation as well – it binds several basic helix-loop-helix factors (bHLH) such as Mash1, NeuroD, Math2, or Nurrr1 and thus regulates the differentiation of neurons [144, 145]. Similar to other members of CIP/KIP family, p57KIP2 possess additional functionality beyond regulation of CDK complexes. The most prominent role is interacting with transcription factors that are required for differentiation and therefore regulating organismal development.

#### Expression and stability regulation

Expression of p57KIP2 is regulated by multiple signals and transcription factors, which reflects its pleiotropic role in differentiation. Transcription of p57KIP2 is positively regulated by E2F1 [146], SP1 [147], HIF-α [148], TGF-β [149], and MyoD through a p73-dependent pathway [150]. On the contrary, Jab1/Csn5 [151] and Hes1 effector of the Notch signaling [152] act as p57KIP2 transcription inhibitors and promote the cell cycle progression. P57KIP1 expression is largely regulated by epigenetic modification. *CDKN1C* promoter contains numerous CpG islands that can be methylated, leading to inhibition of *CDKN1C* transcription, which is one of the main mechanisms of p57KIP2 inactivation in cancer [153]. Additional histone modifications, such as deacetylation and methylation, near the promotor region of p57KIP2, also contribute to negative regulation of its gene expression (153). Major epigenetic modifiers responsible for p57KIP2 downregulation are HDAC1/2 [154], Lsh [155], EZH2 [156], and DNMT3a [157] and inhibition of these enzymes leads to restoration of p57KIP2 expression [147]. On the protein level, p57KIP2 is regulated by proteasomal degradation. Similarly to other members of the CIP/KIP family, the major destruction complex for p57KIP2 is also SCF-Skp2-Cks1 [158], which ubiquitinates p57KIP2 in response to its phosphorylation by Cyclin E/CDK2 complex [158]. Additionally, two F-box proteins bind and target p57KIP2 for SCF-mediated ubiquitination. TGFβ1 stimulates the expression of F-box protein FBL12 in osteoblasts, which results in binding to Threonine 310-phosphorylated p57KIP2 and targets it for degradation [159]. Another F-box protein, FBXO22, is increased in hepatocellular carcinoma or cervical carcinoma, where it promotes cancer progression by binding to p57KIP2 and targeting it to degradation, similar to Fbxo22-mediated p21CIP degradation [160, 161]. Finally, RNF26 was identified as the only non-SCF complex ubiquitin ligase degrading p57KIP2 in response to FoxM1-stimulated RNF26 transcription which is one of the tumor promoting factors in development of bladder carcinoma [162].

#### Posttranslational modifications

Very little is known about the posttranslational modifications of p57KIP2. To date, three phosphorylation sites on p57KIP2 have been described in more detail–Threonine 143, Serine 282, and Threonine 310 (Fig. 4A, B). Ser282 and Thr310 are phosphorylated by Akt, which further strengthens the link between PI3K/Akt signaling and the CIP/KIP family of CKIs [163]. Phosphorylation of p57KIP2 by Akt induces its cytoplasmic localization and subsequent degradation. Regulation of cytoplasmic localization of p57KIP2 by Akt-mediated phosphorylation follows the identical mechanism for other CIP/KIP family members. However, the route to degradation is not understood well, although increased Akt activity decreases the half-life of p57KIP2 [163]. Furthermore, CDKs also phosphorylate p57KIP2 on Threonine 310, which increases its affinity to the SCF complex and stimulates its proteasomal degradation [158]. Finally, p57KIP2 is phosphorylated by major stress-sensing kinase p38 on threonine 143 [139]. As opposed to Akt or CDK phosphorylation, p38-mediated T143 phosphorylation leads to the stabilization of p57KIP2 and increased association with CDK2, resulting in inhibition of the cell cycle progression in response to environmental stress [139]. Interestingly, high throughput experiments identified additional sites on the C-terminus of p57KIP2 (S297, S299, T306) that are dephosphorylated in response to Aurora kinases inhibition, which indicates that p57KIP2 is phosphorylated in mitosis and probably targeted to degradation [164]. To date, the role of p57KIP2 phosphorylation has not been explored to a great depth and only canonical phosphorylations were identified – pro-proliferative signals promote phosphorylation leading to p57KIP2 degradation (T143, S282), and stress signals promote stabilizing phosphorylation (T310) (Fig. 4A).

#### P57KIP2 role in cancer

Scientific evidence on p57KIP2’s role in cancer gathered to date points out that p57KIP2 is a *bona fide* tumor suppressor, and no tumor-promoting function has been assigned. Experiments utilizing p57KIP2 complete knockout mouse model confirmed its major role in regulation of development and cellular differentiation as complete knockout leads to embryonal and perinatal lethality and only 10% of animals reach adulthood [133, 134]. Mice surviving to adulthood however do not exhibit higher rate of spontaneous tumorigenesis [134]. In human cancers, decreased p57KIP2 expression is observed in various types of malignancies, including hepatocellular [151], prostate [165], colorectal [166], pancreatic [167], pulmonary [168], and breast [169], as well as in bladder cancer [170]. Additionally, p57KIP2 expression negatively correlates with tumor aggressiveness and survival [171, 172]. The major mechanism of p57KIP2 downregulation in cancer is epigenetic chromatin modification of *CDKN1C* promoter. *CDKN1C* promoter DNA hypermethylation is the most common mechanisms, and inhibition of DNA methylation indeed increases p57KIP2 expression and impairs tumor growth [173]. Similarly, deposition of methyl marks on H3K27 within *CDKN1C* locus EZH1/2 is another key cancer-promoting epigenetic modification that downregulates p57KIP2 expression. Indeed inhibition of EZH1/2 results in increased p27KIP1 expression and tumor growth inhibition [174]. Finally, p57KIP2 degradation in tumors is increased in response to oncogenic signaling as well. PI3K/Akt pathway is commonly upregulated in tumors, which results in increased p57KIP2 phosphorylation (T143, S282), nuclear export, and degradation [175]. Interestingly, Oka and colleagues identified p57KIP2-positive quiescent cancer stem cells in colorectal cancer responsible for tumor recurrence after chemotherapy. Eradication of p57KIP2 + quiescence cells leads to suppression of tumor regrowth, which indicates possible therapeutic potential [176].

P57KIP2 is the least studied member of CIP/KIP family and its role in cancer has not been elucidated to a satisfactory degree. In general, its anti-proliferative function is well established but other non-canonical functions such as apoptosis regulation or cancer stem cell maintenance deserves more attention.

### INK4 family

The major role of the INK4 protein family, consisting of four Cyclin-dependent kinase inhibitors (CDKI), p16INK4a, p15INK4b, p18INK4c, and p19INK4d, is inhibition of the progression of the cell cycle by directly binding to Cyclin-dependent kinase 4 and 6 (CDK4, CDK6) and inhibiting their function [177]. The first discovered members of the family [178, 179] p16INK4a and p15INK4b are encoded by related homologous genes (*CDKN2A* and *CDKN2B*) located on chromosome 9p21 [180, 181] within the same gene locus. Besides p16INK4a and p15INK4b Cyclin-dependent inhibitors, INK4/ARF locus encodes also tumor suppressor p14^ARF^ (encoded from *CDKN2A* gene using alternative reading frame), which stabilizes p53 through inhibition of MDM2, thus activating p53-induced apoptosis [182]. The remaining members of the INK4 family, p18INK4c and p19INK4c are encoded by genes *CDKN2C* and *CDKN2D* located on chromosomes 1p32.3 and 19p13 [183]. While p16INK4a and p15INK4b consist of four tandem ankyrin repeats, p18INK4c and p19INK4d contain five ankyrin repeats [184]. Proteins of the INK4 family structurally share a similar protein fold, but p18INK4c and p19INK4d share less amino acid sequence homology with p16INK4a, p15INK4b, or each other [180, 185].

**Fig. 5:**
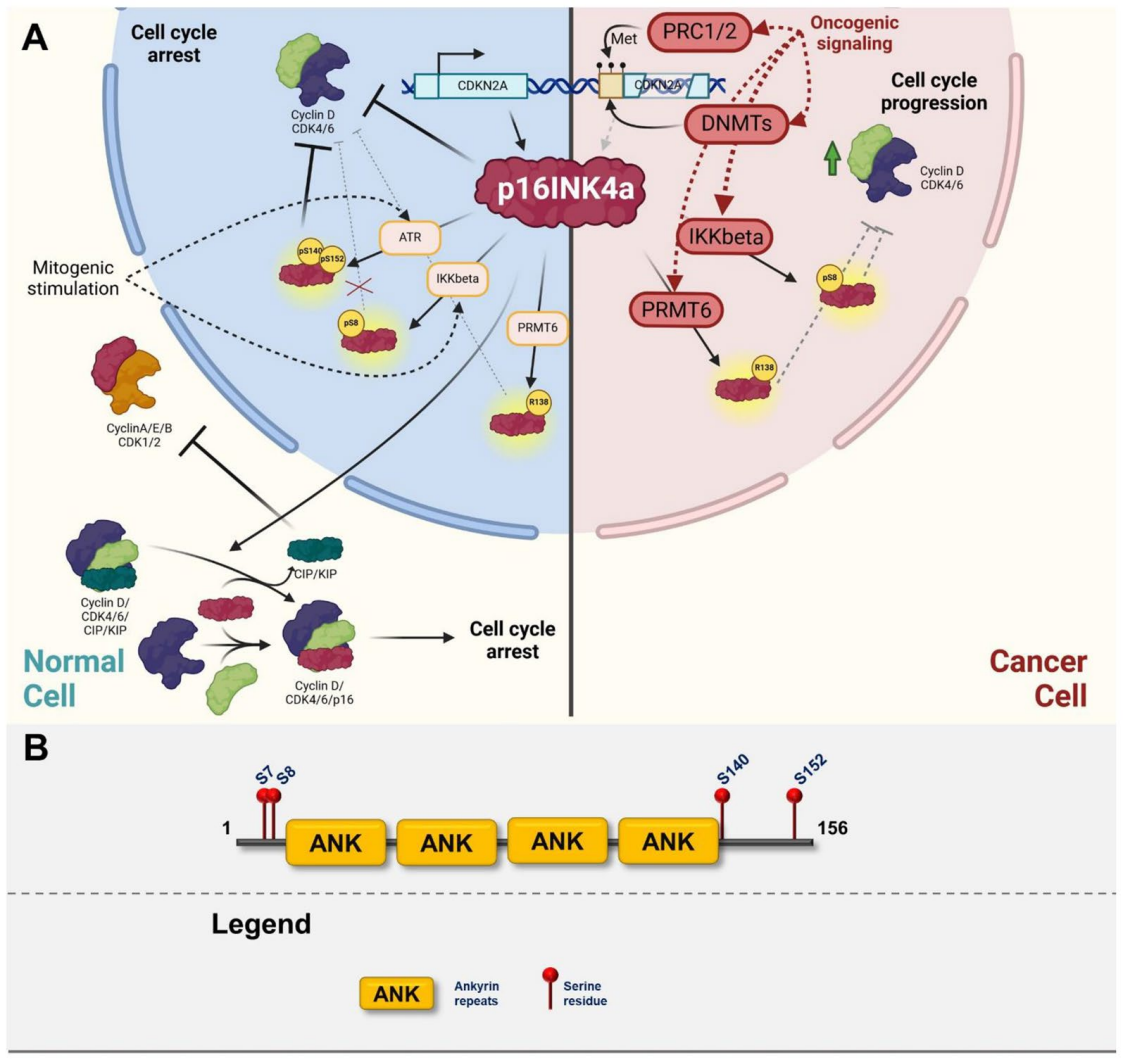
**A** Graphic illustrating the role of p16INK4a in normal cells (left cellular part) and in transformed cells (right cellular part). The graphics focuses on major posttranslational modifications altering the function of p16INK4a and depicting major oncogenic pathways responsible for inactivation or gain-of-function modifications. Created with BioRender.com. **B** p16INK4a domain structure with highlighted sites of posttranslational modifications

**Fig. 6:**
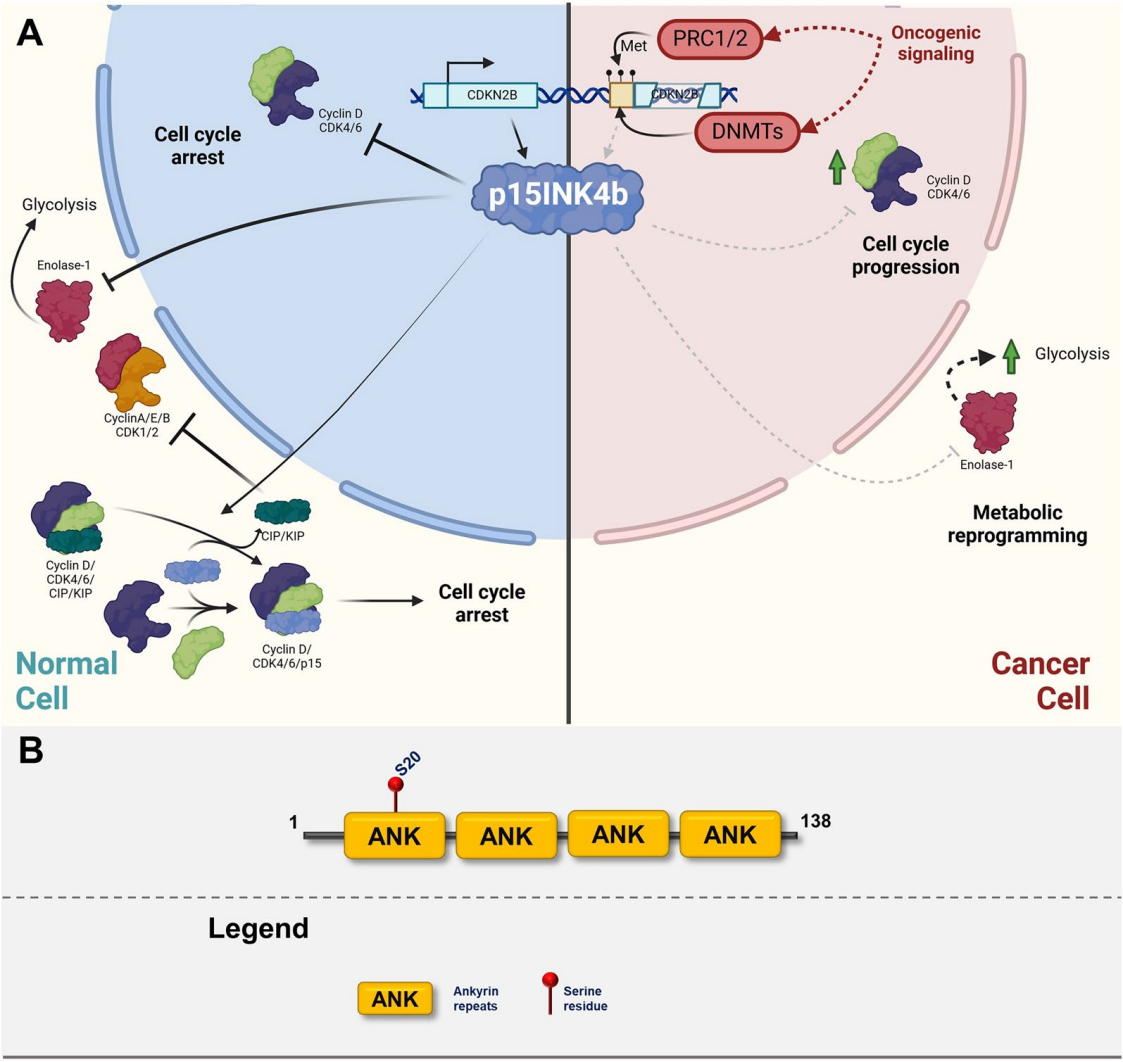
**A** Graphic illustrating the role of p15INK4b in normal cells (left cellular part) and in transformed cells (right cellular part). The graphics focuses on major posttranslational modifications altering the function of p15INK4b and depicting major oncogenic pathways responsible for inactivation or gain-of-function modifications. Created with BioRender.com. **B** p15INK4b domain structure with highlighted sites of posttranslational modifications

**Fig. 7:**
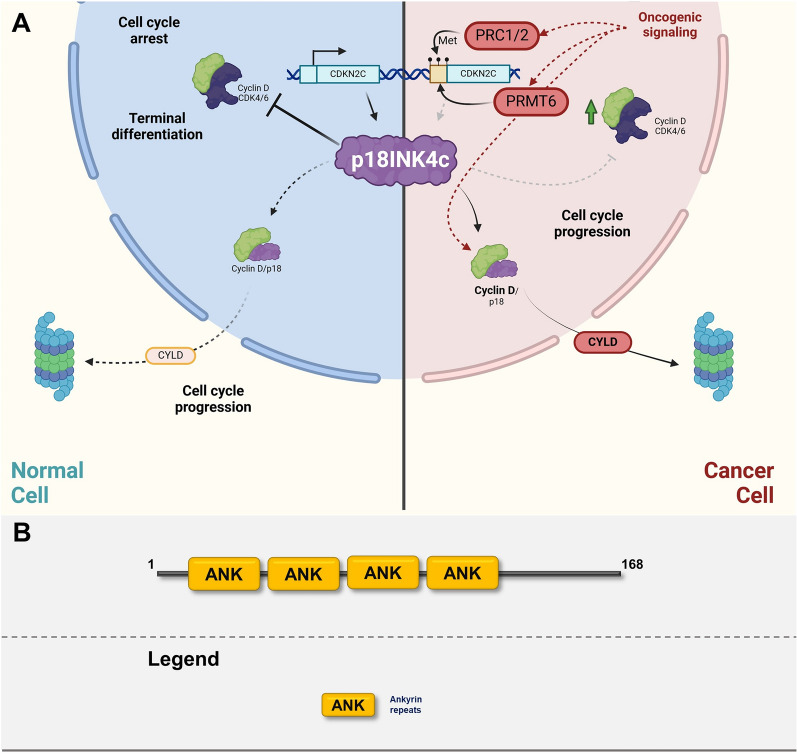
**A** Graphic illustrating the role of p18INK4c in normal cells (left cellular part) and in transformed cells (right cellular part). The graphics focuses on major posttranslational modifications altering the function of p18INK4c and depicting major oncogenic pathways responsible for inactivation or gain-of-function modifications. Created with BioRender.com. **B** p18INK4c domain structure

**Fig. 8:**
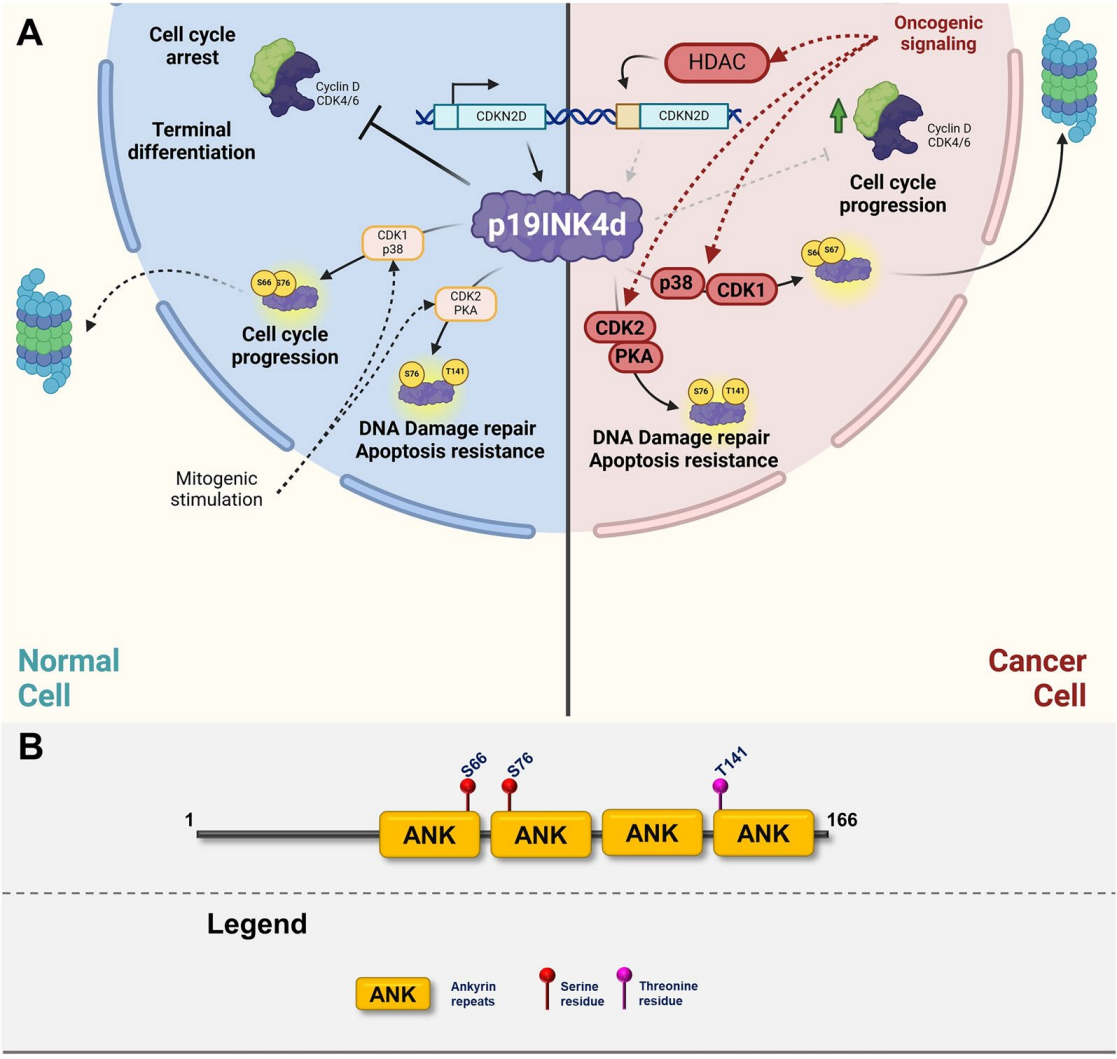
**A** Graphic illustrating the role of p19INK4d in normal cells (left cellular part) and in transformed cells (right cellular part). The graphics focuses on major posttranslational modifications altering the function of p19INK4d and depicting major oncogenic pathways responsible for inactivation or gain-of-function modifications. Created with BioRender.com. **B** p19INK4d domain structure with highlighted sites of posttranslational modifications

INK4 family of Cyclin-depend kinase inhibitors directly binds to CDK4 and CDK6 subunits, which leads to allosteric change, inactivation of their catalytic activity, and inhibition of the formation of CDK-Cyclin complexes [186]. This inhibits downstream phosphorylation of Retinoblastoma protein 1 (Rb1) and subsequently leads to the repression of transcription of S-phase genes and cell cycle arrest [187]. INK4/ARF locus represents a master growth regulator due to its capacity to modulate both proliferation and apoptosis [188] (Figs.  5, 6, 7, 8).

### p16INK4a

#### Function

As stated above, the major role of p16INK4a is the inhibition of CDK4/6 in response to environmental stresses such as DNA damage, oxidative stress, or oncogene overactivation [182, 189]. The primary sides of p16INK4a interactions are helix-turn-helix structures present in the tandem ankyrin repeats. The binding of CDK6 to the cavity of p16INK4a exposes its catalytic cleft inducing an electrostatic interaction between D84 of p16INK4a and R31 of CDK6 (R24 in CDK4), possibly causing a decrease in the kinase activity [190, 191]. Interaction of p16INK4a with CDK4/6 not only directly inhibits CDK4/6 activity but also releases non-INK4 family of CKIs such as p27KIP1 from the complex, allowing them to bind to other Cyclin/CDK complexes (e.g., Cyclin E(A)/CDK2) and inhibit their function [186, 192]. An additional mechanism of the p16INK4a cell cycle inhibition relies on its interaction with the general transcription factor TFIIH. This interaction inhibits the phosphorylation of the carboxyl-terminal domain of the large subunit of RNA polymerase II and downregulates global mRNA transcription [190]. Finally, p16INK4a has been reported to interfere with c-Jun N-terminal kinases 1 and 3 (JNK1 and JNK3) pathways. However, binding of p16INK4a to JNK1/3 doesn’t affect its phosphorylation, rather inhibits downstream phosphorylation of c-Jun which impairs Ras-JNK-Jun-AP-1 signaling [190, 193]. An unbiased proteomic study aiming at the identification of p16INK4a interactors revealed that it interacts with a wide variety of cellular proteins. p16INK4a interacts with PCNA and MCM6, which are proteins that play a role in DNA replication. Furthermore, it interacts with several cytoskeletal proteins (two actin isoforms, four tubulin isoforms, alpha-actin, and myosin regulatory light chain 2), two components of the pre-mRNA splicing machinery of the spliceosome (U1 snRNP A, snRNP-B proteins), chaperones and stress proteins assisting in protein folding. Despite significant p16INK4a interactome, functional characterization of these interactions is completely absent [194]. Since p16INK4a is the major inhibitor of cell cycle progression, it is not surprising that p16INK4a is the key regulator of physiological and pathophysiological senescence. P16INK4a is a biomarker of aging both at the organismal as well as cellular level. p16INK4a expression is increased as a result of various pro-senescence signals (DNA damage, etc.) [195]. Ectopic expression of p16INK4a stimulates cellular senescence, on the other hand, p16INK4a expression inhibition leads to cell cycle reentry and senescence bypass [196]. Importantly, p16INK4a is a key regulator of oncogene-induced senescence, especially in the context of oncogenic activation of the RAF/RAS/ERK pathway [197, 198] and is often deleted or inactivated through promoter hypermethylation in human cancers [199].

#### Regulation of expression and stability

The major mechanism regulating p16INK4a expression is through transcriptional regulation. Since INK4/ARF locus encodes 3 tumor suppressor proteins, it is tightly regulated by numerous transcription factors and epigenetic modifiers. The major transcription factor positively regulating p16INK4a expression is Sp1, which recruits p300 with histone acetyltransferase domain, catalyzing the acetylation of histone H4 [200]. Similarly, Ets1/2 transcription factors activate p16INK4a expression to promote replicative and premature senescence [189]. On the contrary, several transcriptional repressors inhibit p16INK4a transcription. The key factor opposing the role of Ets1 TF is Id1 (inhibitor of DNA binding 1) which indirectly inhibits expression of p16INK4a [189]. Similarly, YY1 transcriptional repressor binds to the p16INK4a promoter and inhibits its transcription and senescence program execution [201]. Moreover, p16INK4a expression is also regulated by the Rb protein through a feedback loop between these two proteins. Phosphorylation of Rb increases p16INK4a expression through the activation of the E2F family of transcriptional factors. Eventually, inhibition of CDK4/6 results in hypo-phosphorylation of Rb and decreased p16INK4a expression [202]. In addition to transcription factors, the regulation of p16INK4a transcription through epigenetic modification has much broader significance. Polycomb repressive complexes (PRC1 and PRC2) are critical for the repression of the locus via catalyzing histone H3 Lys 27 trimethylation. Ectopic expression of PcG (Polycomb group proteins) subunits such as Bmi1, Ezh2, CBX7, and CBX8 leads to the inhibition of p16INK4a expression allowing cells to bypass senescence [196, 203, 204]. On the other hand, ablation of the PcG subunits derepress transcription from the p16INK4a promoter which leads to cell growth inhibition and senescence [196, 204]. Another histone modifier mediating p16INK4a repression is KDM2B histone demethylase. KDM2B demethylates trimethylated H3K4 and di-methylated H3K36, resulting in a decrease in Pol II binding and an increase in trimethylation of H3K27, ultimately leading to the repression of p16INK4a transcription [205, 206]. Finally, p16INK4a expression is inhibited through DNA methylation by the action of DNA methyltransferases (DNMTs). *CDKN2A* was one of the first identified genes that were repressed through methylation of the CpG islands and methylation of its promoter is considered to be one of the earliest events in carcinogenesis [207]. Moreover, genetic ablation of DNMT expression leads to the upregulation of p16INK4a and cell cycle arrest [208, 209]. On the contrary, Jumonji domain-containing D3 protein (JMJD3) is a lysine-specific histone demethylase that demethylates trimethylated H3K27 and acts as a positive regulator of the p16INK4a expression. JMJD3 is activated by Ras-mediated oncogenic signaling which leads to the expression of p16INK4a and entry into oncogene-induced senescence [210, 211].

#### Posttranslational modification

Since p16INK4a is primarily regulated at the transcriptional level, posttranslational modifications have not been well described yet. Similarly, to other CKIs, the major PTM regulating p16INK4a function is phosphorylation. Only 4 phosphorylation sites on p16INK4a have been described to date—Ser7, Ser8, Ser140, and Ser152 (Fig. 5A, B). Phosphorylation of Ser140 by ATR in response to UV stabilizes p16INK4a through impairing Skp2-mediated degradation, and Ser152 phosphorylation greatly increases binding to the Cyclin D/CDK4/6 complexes leading to cell cycle arrest [212, 213]. In contrast, phosphorylation of Ser8 in human fibroblast by IKKβ impairs the interaction of p16INK4a with CDK4 which leads to stimulation of cell cycle and proliferation [214]. Interestingly, another regulatory layer of p16INK4a/CDK interaction was identified–methylation of Arg138 in the close vicinity of Ser140. Methylation of this arginine is mediated by protein arginine methyltransferase 6 (PRMT6) and it has been shown that this methylation interferes with phosphorylation of the Ser140, leading to reduced association with CDK4 [215].

#### Role in cancer

Mutations of *INK4* genes have been associated with a variety of human cancers. Functional studies of gene loss on mouse models can provide valuable information about the role of the *INK4* family of genes in cancer development. Mice lacking one of the tumor suppressors of the INK4 family, generally do not exhibit any major developmental abnormalities. However, these mice are more prone to tumor formation [182, 216, 217]. Mice lacking two *Ink4* genes, such as *Cdkn2a/Cdkn2b* or *Cdkn2a/Cdkn2d*, display increased tumor susceptibility compared to p16INK4a-null mice [182, 218]. Germline mutations that target *Cdkn2a* alone lead to spontaneous tumorigenesis in mice and were linked to familial melanoma [219]. Mutational frequency observed in cancer alters between specific genes of the *INK4* family. The gene with the highest frequency of mutations and deletions in human cancers among this family is *CDKN2A*. *CDKN2B* is often deleted simultaneously with *CDKN2A* [220]. *CDKN2A* is affected in about 10% of all cancers with the highest prevalence of alterations observed in lung adenocarcinoma, pancreatic adenocarcinoma, conventional glioblastoma multiforme, cutaneous melanoma, and bladder urothelial carcinoma [220–224]. Key function attributed to p16INK4a in cancer is strong inhibition of cell cycle leading to senescence that occurs in response to various stress stimuli such as DNA damage or oncogene activation [225]. p16INK4a-mediated senescence leads to repression of genes regulated by the E2F1 transcriptional factor and results in chromatin reorganization [226]. p16INK4a serves as one of the first roadblocks against transformation as it is activated in response to oncogenes and stimulates so called oncogene-induced senescence, especially in the context of overactivation of Ras/MAPK pathway [197, 227]. Expression of p16INK4a and p16INK4a-induced senescence in cancer is also promoted by *TP53* inactivation, accumulation of DNA damage from excessive cell division, and reactive oxygen species accumulation and signaling. It is therefore suggested that p16INK4a serves as a backup for the tumor protein p53 [228]. Since p16INK4a has a strong tumor suppressive functions, its expression is often altered in human cancer. Beyond mutational inactivation, a common mechanism of p16INK4a downregulation is aberrant promoter methylation. Significant hypermethylation was observed in hematological malignancies, cervical cancer, squamous cell carcinoma, ovarian cancer, and multiple myeloma and inhibition of DNA methylation leads to de-repression of p16INK4a expression and growth inhibition [229–233]. As described above, *CDKN2A* inactivation is considered to be one of the earliest events in carcinogenesis [207].

The importance of the INK4 protein family in cancer prognosis and treatment has received limited attention. The level of p16INK4a expression could be used as a biomarker to differentiate early from advanced tumor stages [234]. Methylation of *CDKN2A* promoter was associated with lymph node metastases and large tumor size in primary colorectal cancer tissues [235]. On the other hand, overexpression of p16INK4a in colon adenocarcinomas and breast cancer was linked to poorer prognosis and distant metastasis formation [236, 237]. In head and neck squamous carcinomas p16INK4a-positive and negative circulating tumor cells (CTC) correlate with the survival of patients. Whereas CTCs with the expression of p16INK4a were associated with prolonged progression-free survival and overall survival, p16INK4a-negative CTCs correlated with rapid disease progression after primary chemoradiotherapy [238].

In conclusion, p16INK4a is a key *bona fide* tumor suppressor whose inactivation is present in large amount of human cancers. Since p16INK4a is activated in response to oncogenic transformation, its inactivation is crucial step in transformation cascade and further research into its regulation might bring new approaches for treatment of cancer.

### p15INK4b

#### Function

p15INK4b is closely related to p16INK4a, shares most of its function, and is also transcribed from the same gene locus [239]. p15INK4b is responsible for tissue homeostasis and responds to extracellular growth inhibitory signals, specifically cytokine transforming growth factor-beta (TGF-β) in a variety of cell types [179, 240]. p15INK4b inhibits the cell cycle by binding to CDKs through the N-terminal binding domain. Similar to p16INK4a, it also displaces the CIP/KIP family of CDK inhibitors from the Cyclin D/CDK4/6 complexes, enabling them to act as inhibitors of other CDK-Cyclins [241]. Although it shares a lot of functionality with p16INK4a, a recent study showed that p15INK4b is much more potent in inhibition of carcinogenesis than p16INK4a at least in some context [242]. One study shows that p15INK4b inhibits CDK4/6 activity to a bigger extent than p16INK4a which leads to more profound cell cycle inhibition [242]. Additionally, the study shows that p15INK4b binds to cytoplasmic enolase through the same region as to CDK. Competition between enolase and CDK binding results in the sequestration of p15INK4b in cytoplasm of cells where enolase is overexpressed and activation of CDKs. Moreover, binding of p15INK4b to enolase decreases its activity thus increasing the flux of glucose to the Krebs cycle and inhibiting glycolysis. In conclusion, deletion of p15INK4b in cancer cells leads to a double-trouble situation, where CDKs are overactivated and glycolysis is enhanced which results in high growth promotion [242].

#### Regulation of expression and stability

As previously mentioned, polycomb repressive complexes are critical for the repression of the INK4/ARF locus and subsequent senescence delay via catalyzing histone H3 Lys27 trimethylation [243, 244]. *Kotake *et al*.* showed that long noncoding RNAs contribute to the targeting of PRC2 to the *CDKN2B* locus [245]. ANRIL, long non-coding RNA transcribed in antisense orientation of the INK4/ARF locus, downregulates p15INK4b expression in a variety of cancers and induces cell proliferation [246–249]. Another important negative regulator of p15INK4b is Oct-1 (octamer-binding protein 1), which recruits histone deacetylase complexes to *CDKN2B* promoter and thus inhibits its transcription [250]. In line with this evidence, Oct1 has been shown to promote growth and metastatic dissemination of several types of cancer such as breast, colon or hepatocellular carcinoma [251–253]. Major signaling pathway positively regulating expression of p15INK4b is TGF-β which activates Sp1 and SMAD transcription factors and mediates G1 cell cycle arrest [254, 255]. Finally, similarly to p16INK4a, FOXOs (forkhead box O transcriptional factors) stimulate expression of p15INK4a in the absence of proliferative signals mediated by Akt pathway [256].

#### Posttranslational modifications

In the case of p15INK4b, the range of data about posttranscriptional modifications and their impact on protein binding and activity is limited. The only PTM identified is the phosphorylation of Ser20 which was detected in developing mouse brains using large-scale phosphoproteomic analysis (Fig. 6A, B) [257].

#### Role in cancer

*CDKN2B* mutations independent of INK4/ARF locus mutations are infrequent and have not been well studied [258]. Mice harboring deletion of *CDKN2B* are not prone to tumorogenesis independently of other driver mutations indicating that functionality of other proteins from INK4/Arf locus is sufficient to compensate for p15INK4b deficiency [217, 220]. However, p15INK4b deletion is strongly cooperating with oncogenic Ras (KRAS, HRAS) which leads to emergence of more aggressive lung, pancreatic or bladder tumors then in case of p16INK4a deletion [242, 259, 260]. On the other hand, co-deletion of *CDKN2A* and *CDKN2B* leads to broad spectrum of malignancies [220]. Strong effect of the co-deletion of *Cdkn2a* and *Cdkn2b* on predisposition to the development of a range of highly malignant tumors observed in mice models could be explained by complementing functions of these two proteins. The fact that p15INK4b backs up p16INK4a reinforced the tumor-suppressing capacity of the *INK4/ARF* locus [218]. Although p16INK4a expression can be used as a biomarker for tumor stage grading, because of the distinct role of p16INK4a in tumor suppression and the high frequency of mutations in cancer, Park et al*.* proposed using p15INK4b as an alternative marker for the detection of senescent tumor cells [261, 262]. An interesting observation was made in HRas-promoted urothelial cancer, where the loss of p15INK4b leads not only to the de-repression of cell cycle progression but also to the upregulation of glycolysis through the liberation of enolase from binding to p15INK4b. This observation indicates cytoplasmic function of p15INK4b, similar to the members of the CIP/KIP family, however, it is yet to be determined if this is a general p15INK4b function or specific to the HRas-promoted urothelial cancer. Finally, both p15INK4b and p16INK4a regulate therapy-induced senescence (TIS) of cancer cells. These senescent cells are terminally arrested, however, their tumor-promoting role has been suggested mainly through the secretion of various cytokines that affect the tumor microenvironment–a phenomenon called senescence-associated secretory phenotype–SASP [263, 264]. The plethora of secreted factors includes but is not limited to IL-6, IL-7, IL-8, IL-1a/b, IFN-gamma, EGF, bFGF, uPA, MMP-1/3/10, and many others. These factors play a crucial role in modulating the tumor microenvironment and promoting immune evasion and invasiveness [265, 266]. P16INK4a and p15INK4b are well-established markers of these TIS cells and genetic ablation of p16INK4a indeed impairs SASP and the secretion of IL-6 and IL-8 [267].

Due to the fact that *CDKN2B* is almost always deleted together with *CDKN2A* in human cancer, it has not received sufficient scientific interest. Current evidence shows that although these two members of INK4 family have significantly overlapping roles, there are p15INK4b-unique tumor suppressing function that are illustrated by more aggressive tumors with *CDKN2B* deletion especially in certain signaling context (e.g. oncogenic RAS).

### p18INK4c

To date, p18INK4c received very limited scientific attention in the context of tumorigenesis. p18INK4c is transcribed from the *CDKN2C* gene located on chromosome 1 and is associated with cell cycle arrest in the process of terminal differentiation [268, 269]. Ablation of p18INK4c in mice leads to increased proliferation rate in various organs and tissues, however are prone only to spontaneous development of pituitary tumors later in life [269, 270]. Complete deletion or haploinsufficiency of p18INK4c lead to increased rate and spectrum of tumors induced by chemical carcinogens [271]. Moreover, deletion of p18INK4c has a differential effect in the context of other mutations. For example co-deletion of p18INK4c and p53 lead to development of medulloblastomas, hemangiosarcomas, and other tumors not present in either of the parental strains or deletion of p18INK4c in *PTEN−/−* background resulted in emergence of various tumors such as pituitary or prostate cancers [272, 273]. In human cancers, *CDKN2C* gene deletion or significantly lower mRNA expression is present only in less than 1% of cancers, almost uniquely in low- and high-grade gliomas [58, 59]. At the transcriptional level, p18INK4c is positively regulated by E2F1 and Sp1 transcription factors, forming a negative feedback loop with Rb/E2F1 pathway [274]. On the other hand, p18INK4c expression is negatively regulated through epigenetic modification of its promoter, however there is very limited information. It has been shown that the p18INK4c promoter is hypermethylated in some cases of Hodgkin lymphoma and gastric cancer which leads to the absence of p18INK4c protein expression [275, 276]. More is known about histone modification-mediated regulation of p18INK4c expression. Similar to INK4/ARF locus, the p18INK4c promoter is subject to H3K27 trimethylation which recruits PRC1 and represses transcription of p18INK4c [277, 278]. Oncogenic PRMT6 promotes H3R2 di-methylation which antagonizes H3K4 trimethylation leading to repression of p18INK4c expression [279] (Fig. 7A, B). Much less is known about the protein stability of p18INK4c. It has been shown that p18INK4c is preferentially ubiquitinylated at Lys46 and Lys112 residues and the process of p18INK4c ubiquitination is inhibited by CDK4/6, which leads to protein stabilization. Cyclin D1 accelerates p18INK4c turnover by competing for binding to CDKs [280]. Additionally, CYLD deubiquitinase is removing K48 polyubiquitin chains therefore stabilizing p18INK4c [281]. As mentioned earlier, the *CDKN2C* gene is not regularly inactivated by deletion or point mutations in human cancer, however, loss of p18INK4c expression has been linked to the development of medulloblastomas, hepatocellular cancer, testicular cancer, and medullary thyroid carcinoma [282–286] and promotor hypermethylation of *CDKN2C* has been reported in gastric cancer and Hodgkin lymphomas [275, 287]. Although no non-canonical tumor-promoting role of p18INK4c was identified, p18INK4c is implicated in contributing to resistance to CDK4/6 inhibitors. In abemaciclib-resistant breast cancer, p18INK4c is associated with CDK6 but not with CDK4 and impairs binding of abemaciclib to CDK6 thus rendering it active. Moreover, knockout of p18INK4c partially restored sensitivity of these cells to abemaciclib, and double knockout of p18INK4c and p15INK4b restored sensitivity almost completely [288]. Similarly, in acute myeloid leukemia (AML) the efficacy of CDK6-targeted degrader is also impaired by p18INK4c and p16INK4a [289].

P18INK4c is the least studied member of INK4 family with infrequent mutations in human cancers, but downregulation of its expression through methylation is linked to development of various malignancies. More recent evidence shows that it plays a role in resistance to CDK6-targeted therapies which warrant closer investigation of its functionality.

### p19INK4d

#### Function

p19INK4d is one of the more studied INK4 family members and several functions have been assigned to it. Beyond regulating the cell cycle, it plays a role in the regulation of apoptosis, DNA damage repair, and senescence. Similarly, to other family members, p19INK4d directly binds to Cyclin-dependent kinases 4 and 6 and induces cell cycle arrest [177]. However, knockout experiments in mice showed that deletion of p19INK4d is not associated with a higher prevalence of tumors, rather it impairs terminal development of certain tissues such as male reproductive tissue or central nervous system neurons [290, 291]. Similarly, it has been shown that p19INK4d also modulates GATA1 protein levels in human terminal erythropoiesis through a novel pathway, involving phosphatidylethanolamine-binding protein 1 (PEBP1), phosphorylated extracellular signal-regulated kinase (pERK), and heat shock 70 kDa protein (HSP70) [292]. Another cell cycle-related function is the regulation of senescence. P19INK4d is upregulated in response to senescence-inducing signaling resulting in increased deposition of p19INK4d on chromatin and increased global chromatin heterochromatization characteristic for senescent cells [293]. Similar to other CKIs, it has been shown that p19INK4d is also regulating DNA damage repair. P19INK4d is upregulated upon genotoxic stress and promotes cell survival and DNA damage repair through binding to chromatin and increasing the accessibility of damaged DNA to repair machinery [294–296].

#### Regulation of expression and stability

Expression of p19INK4d is mainly regulated on the transcriptional level. One of the key regulatory interactions is E2F1-mediated p19INK4d periodic expression throughout the cell cycle. E2F1 promotes the expression of p19INK4d at the end of the G1 phase which leads to the inhibition of CDK4/6 and the transition to the S-phase of the cell cycle [297]. Similarly, E2F1 also stimulates the expression of p19INK4d in response to DNA damaging agents which results in cell cycle arrest [298]. Expression of p19INK4d is also induced by forkhead box O transcriptional factors (FOXOs), which are involved in the induction of G1 arrest caused by Akt signaling inactivation [256]. ER8 element in the promotor region of p19INK4d is recognized as a response element of the retinoic acid (RA) receptor/ retinoid X receptor heterodimer and the vitamin D3 receptor/ retinoid X receptor heterodimer. Induction of p19INK4d by RA signaling leads to decreased autophagic cell death [299]. In acute promyelocytic leukemia, chimeric PML-RARα protein blocks the binding sides of RA receptors, which leads to the inhibition of p19INK4d expression, inhibition of normal RA receptor signaling, and senescence [300]. Similar to other INK4 family members, p19INK4d expression is regulated through epigenetic modifications. P19INK4d promoter is a target of HDAC1/2 which inhibits its expression and pharmacological and transcriptional inhibition of HDAC1/2 leads to upregulation of p19INK4d and cell cycle arrest [301]. On the other hand, there is only limited information on p19INK4d degradation. P19INK4d is a highly unstable protein especially in the G0/G1 phase of the cell cycle with a half-life of 20–30 min [302, 303]. It is well established that the interaction of p19INK4d with CDK4/6 leads to its increased degradation and it depends on the integrity of lysine 62 which is the main site for p19INK4d ubiquitination [302, 304]. Although the ubiquitin ligase responsible for p19INK4d has not been determined yet, the APC/C-Cdh1 complex would be a prime candidate because of its high activity throughout the G1 phase [305].

Regulation of p19INK4d expression and stability follows the canonical route where its increased through environmental stress-mediated signaling (DNA damage) or at the G1/S transition stage when it’s necessary to inhibit CDK4/activity, and is decreased in response to pro-proliferative signals (Akt activation).

#### Posttranslational modification

There are 3 reported phosphorylation sites on the p19INK4d molecule–Serine 66, 76, and Threonine 141 (Fig. 8A, B). Reports investigating the interplay between the phosphorylation of these sites, protein stability, and binding partners show contradictory results. p19INK4d is sequentially phosphorylated by p38 and CDK1 on serine residues 66 and 76 leading to the local unfolding of the protein structure and dissociation of the p19INK4d-CDK complex. The locally unfolded protein then undergoes ubiquitination and eventual degradation [304]. The evidence supporting the role of these phosphorylations is quite compelling but it’s in contrast with increased p19INK4d protein levels in S and G2 phases where CDK1 would be the most active. Site-directed mutagenesis and chemical synthesis experiments confirm that p38-directed S66 phosphorylation alone doesn’t increase p19INK4d degradation, but primes it for phosphorylation by CDK1 and double phosphorylated p19INK4d is targeted to proteasome-mediated degradation [304, 306]. Another signal triggering Ser76 phosphorylation is genotoxic stress. Phosphorylation of Ser76 is mediated by CDK2, which subsequently stimulates phosphorylation of Thr141 by PKA. Both of these phosphorylations are indispensable for p19INK4d´s role in the stimulation of DNA damage repair and apoptosis protection [307]. These contradictory results could be reconciled by postulating the existence of different fractions of p19INK4d that are differentially phosphorylated, depending on the cellular context and possibly the subcellular localization of p19INK4d.

#### Role in cancer

In vivo mouse experiments established p19INK4d as a canonical tumor suppressor as mice having a complete deletion of *CDKN2D* gene showed increased frequency of development of wide range of tumors such as pituitary and lung adenomas, lymphomas, hemangiosarcoma, thyroid cancer, and insulinoma [308].

However, *CDKN2D* alterations are very rare in human cancers, and according to the TCGA database, the rate is below 1%. Moreover, there is no directionality towards deletion, inactivating mutations, or promoter hypermethylation, as one would expect for canonical tumor suppressors [58, 59]. Overall, there is very limited information gathered on the role of p19INK4d in human cancers, and in general, there is no clear association with the outcome, with a few exceptions. For example, in ovarian cancer, p19INK4d protein expression is negatively correlated with prognosis and survival especially in p53-deficient tumors [309]. On the other hand, the role of p19INK4d in hepatocellular carcinoma is opposite and it has been shown that the loss of p19INK4d expression correlates with tumor aggressiveness [310]. Based on the low prevalence of p19INK4d alterations in cancer and on the functional evidence we could conclude that the role of p19INK4d might be context-dependent and could play a role in resistance to genotoxic treatment and to CDK4/6-inhibitors.

## Conclusions and future perspectives

Initially, the function of CIP/KIP and INK4 protein families was considered to be the inhibition of cell cycle progression, but growing evidence shows that these proteins possess multiple other cell cycle-dependent as well as cell cycle-independent roles. It has been shown that beyond binding and altering the function of Cyclin/CDK complexes, they affect gene expression, apoptosis and invasiveness. In non-transformed cells they work as *bona fide* tumor suppressors, however oncogenic signaling alters their function to promote hallmarks of cancer such as apoptosis resistance, DNA damage repair or metastasis formation. Therefore, a deeper understanding of how oncogenic signaling pathways hijack these proteins and how to revert it could support novel therapeutic approaches especially in conjunction with the existing ones. Key areas of investigation could include but not be limited to:

o How to inhibit p21CIP T145/S146 phosphorylation and/or ensure retention of T145/S146 phosphorylated p21CIP in nucleus.
o How to disrupt p21CIP and p27KIP1 scaffolding functions to impair formation of Cyclin D/CDK4/6 complexes.
o How to prevent cytoplasmic localization of p27KIP1 and conversely promote stability and binding of p27KIP1 co Cyclin A(E/B)/CDK1[2] complexes.
o How to stably increase expression of the INK4 family through epigenetic activation of the promoters.
o How to block S8 phosphorylation and R138 methylation on p16INK4a to increase its association with CDKs.
o Is there a possibility for synthetic lethality in cancer types with deletion of p16INK4a and p15INK4b?
o How to counteract INK4-mediated resistance to CDK4/6 inhibitors?

Additionally, above mentioned lines of research could be investigated in the context of current state-of-art pharmacological inhibitors. At the moment, there are 3 highly specific CDK4/6 inhibitors approved for treatment of hormone receptor positive (HR^+^), HER2 negative (HER2^−^) advanced and metastatic breast cancer (Palbociclib, Abemaciclib, Ribociclib) [311–313] and one approved as a supportive, myeloprotective therapy for non-small cell lung cancer (Trilaciclib) [314]. Although these inhibitors are only approved for a subset of cancer types, they have been investigated in clinical trials for treatment of multiple malignancies such as ovarian cancer, pancreatic ductal adenocarcinoma, colorectal cancer, non-small cell lung cancer, endometrial cancer, head and neck squamous cell carcinoma and others [315]. Although these inhibitors are highly specific and have favorable toxicity profile, intrinsic and acquired resistance is a major challenge. In clinic, only a smaller portion of patient respond to these inhibitors and even responders eventually develop resistance [316]. Multiple mechanisms of resistance have been described such as Rb1 mutations, CCND1 and CCNE1 amplification, or activation of parallel pathways such as FGFR or PI3K/Akt pathways or paradoxically amplification of INK4-coding genes [317, 318]. Therefore there is a great need to identify additional biomarkers or synthetically lethal combinations with CDK4/6 inhibitors.

Impairing function of CIP/KIP and INK4 families in specific compartments (cytoplasm) could represent one of the research avenues that could advance the use of current pharmacological CDK4/6 inhibitors. As the function of cellular CKIs is regulated by their posttranslational modifications, understanding how these could be targeted might aid in development therapies that would complement the use of CDK4/6 inhibitor and expand their efficacies to other tumor types beyond HR^+^ HER2^−^ breast cancer.

## Data Availability

Not applicable.

## References

[1] Schirripa A, Sexl V, Kollmann K (2022). Cyclin-dependent kinase inhibitors in malignant hematopoiesis. Front Oncol.

[2] LaBaer J, Garrett MD, Stevenson LF, Slingerland JM, Sandhu C, Chou HS (1997). New functional activities for the p21 family of CDK inhibitors. Genes Dev.

[3] Abbas T, Dutta A (2009). p21 in cancer: intricate networks and multiple activities. Nat Rev Cancer.

[4] Larrea MD, Liang J, Da Silva T, Hong F, Shao SH, Han K (2008). Phosphorylation of p27 ^Kip1^ regulates assembly and activation of cyclin D1-Cdk4. Mol Cell Biol.

[5] Bagui TK, Jackson RJ, Agrawal D, Pledger WJ (2000). Analysis of cyclin D3-cdk4 complexes in fibroblasts expressing and lacking p27 ^*kip1*^ and p21 ^*cip1*^. Mol Cell Biol.

[6] Huang Y, Yoon MK, Otieno S, Lelli M, Kriwacki RW (2015). The activity and stability of the intrinsically disordered Cip/Kip protein family AreRegulated by Non-Receptor TyrosineKinases. J Mol Biol.

[7] Kriwacki RW, Hengst L, Tennant L, Reed SI, Wright PE (1996). Structural studies of p21Waf1/Cip1/Sdi1 in the free and Cdk2-bound state: conformational disorder mediates binding diversity. Proc Natl Acad Sci.

[8] Adkins JN, Lumb KJ (2002). Intrinsic structural disorder and sequence features of the cell cycle inhibitor p57 ^Kip2^. Prote Struct Funct Bioinformat..

[9] Bienkiewicz EA, Adkins JN, Lumb KJ (2002). Functional Consequences of Preorganized Helical Structure in the Intrinsically Disordered Cell-Cycle Inhibitor p27 ^Kip1^. Biochemistry.

[10] Baker SJ, Reddy EP (2012). CDK4: A Key Player in the Cell Cycle, Development, and Cancer. Genes Cancer.

[11] Li Y, Jenkins CW, Nichols MA, Xiong Y (1994). Cell cycle expression and p53 regulation of the cyclin-dependent kinase inhibitor p21. Oncogene.

[12] Baus F, Gire V, Fisher D, Piette J, Dulić V (2003). Permanent cell cycle exit in G2 phase after DNA damage in normal human fibroblasts. EMBO J.

[13] Georgakilas AG, Martin OA, Bonner WM (2017). p21: a two-faced genome guardian. Trends Mol Med.

[14] Parveen A, Akash MSH, Rehman K, Kyunn WW (2016). Dual role of p21 in the progression of cancer and its treatment. Crit Rev Eukaryot Gene Expr.

[15] Wade HJ (1993). The p21 Cdk-interacting protein Cip1 is a potent inhibitor of G1 cyclin-dependent kinases. Cell.

[16] Saha P, Eichbaum Q, Silberman ED, Mayer BJ, Dutta A (1997). p21 ^*CIP1*^ and Cdc25A: competition between an inhibitor and an activator of cyclin-dependent kinases. Mol Cell Biol.

[17] Smits VAJ, Klompmaker R, Vallenius T, Rijksen G, Mäkelä TP, Medema RH (2000). p21 inhibits Thr161 phosphorylation of Cdc2 to enforce the G2 DNA damage checkpoint. J Biol Chem.

[18] Cheng M (1999). The p21Cip1 and p27Kip1 CDK `inhibitors’ are essential activators of cyclin D-dependent kinases in murine fibroblasts. EMBO J.

[19] Guiley KZ, Stevenson JW, Lou K, Barkovich KJ, Kumarasamy V, Wijeratne TU (2019). p27 allosterically activates cyclin-dependent kinase 4 and antagonizes palbociclib inhibition. Science.

[20] Tom S, Ranalli TA, Podust VN, Bambara RA (2001). Regulatory roles of p21 and Apurinic/Apyrimidinic endonuclease 1 in base excision repair. J Biol Chem.

[21] Koike M, Yutoku Y, Koike A (2011). Accumulation of p21 proteins at DNA damage sites independent of p53 and core NHEJ factors following irradiation. Biochem Biophys Res Commun.

[22] Mauro M, Rego MA, Boisvert RA, Esashi F, Cavallo F, Jasin M (2012). p21 promotes error-free replication-coupled DNA double-strand break repair. Nucleic Acids Res.

[23] Jung YS, Qian Y, Chen X (2010). Examination of the expanding pathways for the regulation of p21 expression and activity. Cell Signal.

[24] Karimian A, Ahmadi Y, Yousefi B (2016). Multiple functions of p21 in cell cycle, apoptosis and transcriptional regulation after DNA damage. DNA Repair.

[25] Gartel AL, Goufman E, Najmabadi F, Tyner AL (2000). Sp1 and Sp3 activate p21 (WAF1/CIP1) gene transcription in the Caco-2 colon adenocarcinoma cell line. Oncogene.

[26] Karkhanis M, Park JI (2015). Sp1 regulates Raf/MEK/ERK-induced p21(CIP1) transcription in TP53-mutated cancer cells. Cell Signal.

[27] Pardali K, Kurisaki A, Morén A, ten Dijke P, Kardassis D, Moustakas A (2000). Role of Smad proteins and transcription factor Sp1 in p21(Waf1/Cip1) regulation by transforming growth factor-beta. J Biol Chem.

[28] Elston R, Inman GJ (2012). Crosstalk between p53 and TGF- <math> <mi mathvariant="bold">β</mi> </math> Signalling. J Signal Transduct.

[29] Decesse JT, Medjkane S, Datto MB, Crémisi CE (2001). RB regulates transcription of the p21/WAF1/CIP1 gene. Oncogene.

[30] Gartel AL, Tyner AL (1999). Transcriptional regulation of the p21(WAF1/CIP1)Gene. Exp Cell Res.

[31] Xu H, Wang Z, Jin S, Hao H, Zheng L, Zhou B (2014). Dux4 induces cell cycle arrest at G1 phase through upregulation of p21 expression. Biochem Biophys Res Commun.

[32] Nishitani H, Shiomi Y, Iida H, Michishita M, Takami T, Tsurimoto T (2008). CDK inhibitor p21 Is degraded by a proliferating cell nuclear antigen-coupled Cul4-DDB1Cdt2 pathway during S phase and after UV irradiation. J Biol Chem.

[33] Abbas T, Sivaprasad U, Terai K, Amador V, Pagano M, Dutta A (2008). PCNA-dependent regulation of p21 ubiquitylation and degradation via the CRL4 ^Cdt2^ ubiquitin ligase complex. Genes Dev.

[34] Wang W, Nacusi L, Sheaff RJ, Liu X (2005). Ubiquitination of p21 Cip1/WAF1 by SCF Skp2: substrate requirement and ubiquitination site selection. Biochemistry.

[35] Yu ZK, Gervais JLM, Zhang H (1998). Human CUL-1 associates with the SKP1/SKP2 complex and regulates p21 ^CIP1/WAF1^ and cyclin D proteins. Proc Natl Acad Sci.

[36] Bornstein G, Bloom J, Sitry-Shevah D, Nakayama K, Pagano M, Hershko A (2003). Role of the SCFSkp2 Ubiquitin Ligase in the degradation of p21Cip1 in S Phase. J Biol Chem.

[37] Amador V, Ge S, Santamaría PG, Guardavaccaro D, Pagano M (2007). APC/C(Cdc20) controls the ubiquitin-mediated degradation of p21 in prometaphase. Mol Cell.

[38] Deng T, Yan G, Song X, Xie L, Zhou Y, Li J (2018). Deubiquitylation and stabilization of p21 by USP11 is critical for cell-cycle progression and DNA damage responses. Proc Natl Acad Sci.

[39] Hwang CY, Lee C, Kwon KS (2009). Extracellular signal-regulated kinase 2-dependent phosphorylation induces cytoplasmic localization and degradation of p21 ^Cip1^. Mol Cell Biol.

[40] Rössig L, Badorff C, Holzmann Y, Zeiher AM, Dimmeler S (2002). Glycogen synthase kinase-3 couples AKT-dependent signaling to the regulation of p21Cip1 degradation. J Biol Chem.

[41] Densham RM, O’Neill E, Munro J, König I, Anderson K, Kolch W (2009). MST kinases monitor actin cytoskeletal integrity and signal via c-Jun N-terminal kinase stress-activated kinase to regulate p21 ^Waf1/Cip1^ stability. Mol Cell Biol.

[42] Kim GY, Mercer SE, Ewton DZ, Yan Z, Jin K, Friedman E (2002). The stress-activated protein kinases p38α and JNK1 stabilize p21Cip1 by phosphorylation. J Biol Chem.

[43] Zhu H, Nie L, Maki CG (2005). Cdk2-dependent Inhibition of p21 stability via a C-terminal cyclin-binding motif. J Biol Chem.

[44] Järviluoma A, Child ES, Sarek G, Sirimongkolkasem P, Peters G, Ojala PM (2006). Phosphorylation of the cyclin-dependent kinase inhibitor p21 ^Cip1^ on serine 130 is essential for viral cyclin-mediated bypass of a p21 ^Cip1^ -imposed G _1_ arrest. Mol Cell Biol.

[45] Li Y, Dowbenko D, Lasky LA (2002). AKT/PKB phosphorylation of p21Cip/WAF1 enhances protein stability of p21Cip/WAF1 and promotes cell survival. J Biol Chem.

[46] Zhou BP, Liao Y, Xia W, Spohn B, Lee MH, Hung MC (2001). Cytoplasmic localization of p21Cip1/WAF1 by Akt-induced phosphorylation in HER-2/neu-overexpressing cells. Nat Cell Biol.

[47] Zhang Y, Wang Z, Magnuson NS (2007). Pim-1 Kinase-dependent phosphorylation of p21Cip1/WAF1 regulates its stability and cellular localization in H1299 cells. Mol Cancer Res.

[48] Wang Z, Zhang Y, Gu JJ, Davitt C, Reeves R, Magnuson NS (2010). Pim-2 phosphorylation of p21Cip1/WAF1 enhances its stability and inhibits cell proliferation in HCT116 cells. Int J Biochem Cell Biol.

[49] Suzuki H, Yabuta N, Okada N, Torigata K, Aylon Y, Oren M (2013). Lats2 phosphorylates p21/CDKN1A after UV irradiation and regulates apoptosis. J Cell Sci.

[50] Nakakido M, Deng Z, Suzuki T, Dohmae N, Nakamura Y, Hamamoto R (2015). PRMT6 increases cytoplasmic localization of p21CDKN1A in cancer cells through arginine methylation and makes more resistant to cytotoxic agents. Oncotarget.

[51] García-Fernández RA, García-Palencia P, Sánchez MÁ, Gil-Gómez G, Sánchez B, Rollán E (2011). Combined loss of p21(waf1/cip1) and p27(kip1) enhances tumorigenesis in mice. Lab Invest.

[52] Martín-Caballero J, Flores JM, García-Palencia P, Serrano M (2001). Tumor susceptibility of p21(Waf1/Cip1)-deficient mice. Cancer Res.

[53] Poole AJ, Heap D, Carroll RE, Tyner AL (2004). Tumor suppressor functions for the Cdk inhibitor p21 in the mouse colon. Oncogene.

[54] Stewart ZA, Mays D, Pietenpol JA (1999). Defective G1-S cell cycle checkpoint function sensitizes cells to microtubule inhibitor-induced apoptosis. Cancer Res.

[55] Wendt J, Radetzki S, von Haefen C, Hemmati PG, Güner D, Schulze-Osthoff K (2006). Induction of p21CIP/WAF-1 and G2 arrest by ionizing irradiation impedes caspase-3-mediated apoptosis in human carcinoma cells. Oncogene.

[56] Sohn D, Essmann F, Schulze-Osthoff K, Jänicke RU (2006). p21 blocks irradiation-induced apoptosis downstream of mitochondria by inhibition of cyclin-dependent kinase-mediated caspase-9 activation. Cancer Res.

[57] Herůdková J, Paruch K, Khirsariya P, Souček K, Krkoška M, Vondálová Blanářová O (2017). Chk1 inhibitor SCH900776 effectively potentiates the cytotoxic effects of platinum-based chemotherapeutic drugs in human colon cancer cells. Neoplasia.

[58] Cerami E, Gao J, Dogrusoz U, Gross BE, Sumer SO, Aksoy BA (2012). The cBio cancer genomics portal: an open platform for exploring multidimensional cancer genomics data. Cancer Discov.

[59] Gao J, Aksoy BA, Dogrusoz U, Dresdner G, Gross B, Sumer SO (2013). Integrative analysis of complex cancer genomics and clinical profiles using the cBioPortal. Sci Signal..

[60] Chen Z, Wang K, Hou C, Jiang K, Chen B, Chen J (2017). CRL4BDCAF11 E3 ligase targets p21 for degradation to control cell cycle progression in human osteosarcoma cells. Sci Rep.

[61] Wang Y, Yan F, Nasar A, Chen ZS, Altorki NK, Stiles B (2021). CUL4high lung adenocarcinomas are dependent on the CUL4-p21 ubiquitin signaling for proliferation and survival. Am J Pathol.

[62] Fan T, Jiang S, Chung N, Alikhan A, Ni C, Lee CCR (2011). EZH2-dependent suppression of a cellular senescence phenotype in melanoma cells by inhibition of p21/ *CDKN1A* expression. Mol Cancer Res.

[63] Vincent AJ, Ren S, Harris LG, Devine DJ, Samant RS, Fodstad O (2012). Cytoplasmic translocation of p21 mediates NUPR1-induced chemoresistance. FEBS Lett.

[64] Suzuki A, Tsutomi Y, Yamamoto N, Shibutani T, Akahane K (1999). Mitochondrial regulation of cell death: mitochondria are essential for procaspase 3–p21 complex formation to resist fas-mediated cell death. Mol Cell Biol.

[65] Xia X, Ma Q, Li X, Ji T, Chen P, Xu H (2011). Cytoplasmic p21 is a potential predictor for cisplatin sensitivity in ovarian cancer. BMC Cancer.

[66] Xia X, Ji T, Liu R, Weng Y, Fang Y, Wang Z (2015). Cytoplasmic p21 is responsible for paclitaxel resistance in ovarian cancer A2780 cells. Eur J Gynaecol Oncol.

[67] Maiuthed A, Ninsontia C, Erlenbach-Wuensch K, Ndreshkjana B, Muenzner J, Caliskan A (2018). Cytoplasmic p21 mediates 5-fluorouracil resistance by inhibiting Pro-apoptotic Chk2. Cancers.

[68] Vitiello PF, Staversky RJ, Gehen SC, Johnston CJ, Finkelstein JN, Wright TW (2006). p21Cip1 protection against hyperoxia requires Bcl-XL and is uncoupled from its ability to suppress growth. Am J Pathol.

[69] Vitiello P, Wu Y, Staversky R, Oreilly M (2009). p21Cip1 protects against oxidative stress by suppressing ER-dependent activation of mitochondrial death pathways. Free Radic Biol Med.

[70] Giovannini C, Baglioni M, Toaldo MB, Ventrucci C, D’Adamo S, Cipone M (2013). Notch3 inhibition enhances sorafenib cytotoxic efficacy by promoting GSK3β phosphorylation and p21 down-regulation in hepatocellular carcinoma. Oncotarget.

[71] Fu T, Ma X, Du SL, Ke ZY, Wang XC, Yin HH (2023). p21 promotes gemcitabine tolerance in A549 cells by inhibiting DNA damage and altering the cell cycle. Oncol Lett.

[72] Cheng T, Rodrigues N, Shen H, Yang YG, Dombkowski D, Sykes M (2000). Hematopoietic stem cell quiescence maintained by p21cip1/waf1. Science.

[73] Toyoshima H, Hunter T (1994). p27, a novel inhibitor of G1 cyclin-Cdk protein kinase activity, is related to p21. Cell.

[74] Abbastabar M, Kheyrollah M, Azizian K, Bagherlou N, Tehrani SS, Maniati M (2018). Multiple functions of p27 in cell cycle, apoptosis, epigenetic modification and transcriptional regulation for the control of cell growth: a double-edged sword protein. DNA Repair.

[75] Fero ML, Rivkin M, Tasch M, Porter P, Carow CE, Firpo E (1996). A syndrome of multiorgan hyperplasia with features of gigantism, tumorigenesis, and female sterility in p27Kip1-deficient mice. Cell.

[76] Kiyokawa H, Kineman RD, Manova-Todorova KO, Soares VC, Huffman ES, Ono M (1996). Enhanced growth of mice lacking the cyclin-dependent kinase inhibitor function of p27Kip1. Cell.

[77] Nakayama K, Ishida N, Shirane M, Inomata A, Inoue T, Shishido N (1996). Mice lacking p27(Kip1) display increased body size, multiple organ hyperplasia, retinal dysplasia, and pituitary tumors. Cell.

[78] Bencivenga D, Stampone E, Roberti D, Della Ragione F, Borriello A (2021). p27Kip1, an intrinsically unstructured protein with scaffold properties. Cells.

[79] Rath SL, Senapati S (2016). Mechanism of p27 unfolding for CDK2 reactivation. Sci Rep.

[80] Grimmler M, Wang Y, Mund T, Cilenšek Z, Keidel EM, Waddell MB (2007). Cdk-inhibitory activity and stability of p27 are directly regulated by oncogenic tyrosine kinases. Cell.

[81] Bagui TK, Mohapatra S, Haura E, Pledger WJ (2007). p27 ^Kip1^ and p21 ^Cip1^ are not required for the formation of active D cyclin-cdk4 complexes. Mol Cell Biol.

[82] Ou 欧 力 L, Ferreira AM, Otieno S, Xiao 肖 利民 L, Bashford D, Kriwacki RW. Incomplete Folding upon Binding Mediates Cdk4/Cyclin D Complex Activation by Tyrosine Phosphorylation of Inhibitor p27 Protein. Journal of Biological Chemistry. 2011;286(34):30142–51. 10.1074/jbc.M111.244095 PMC3191053 21715330

[83] Pippa R, Espinosa L, Gundem G, García-Escudero R, Dominguez A, Orlando S (2012). p27Kip1 represses transcription by direct interaction with p130/E2F4 at the promoters of target genes. Oncogene.

[84] Perearnau A, Orlando S, Islam AB, Gallastegui E, Martínez J, Jordan A (2017). p27Kip1, PCAF and PAX5 cooperate in the transcriptional regulation of specific target genes. Nucleic Acids Res.

[85] Yoon H, Kim M, Jang K, Shin M, Besser A, Xiao X (2019). p27 transcriptionally coregulates cJun to drive programs of tumor progression. Proc Natl Acad Sci.

[86] Zhao D, Besser AH, Wander SA, Sun J, Zhou W, Wang B (2015). Cytoplasmic p27 promotes epithelial–mesenchymal transition and tumor metastasis via STAT3-mediated Twist1 upregulation. Oncogene.

[87] Besson A, Gurian-West M, Schmidt A, Hall A, Roberts JM (2004). p27 ^Kip1^ modulates cell migration through the regulation of RhoA activation. Genes Dev.

[88] Bencivenga D, Tramontano A, Borgia A, Negri A, Caldarelli I, Oliva A (2014). p27Kip1 serine 10 phosphorylation determines its metabolism and interaction with cyclin-dependent kinases. Cell Cycle.

[89] Campos T, Ziehe J, Palma M, Escobar D, Tapia JC, Pincheira R (2016). Rheb promotes cancer cell survival through p27Kip1-dependent activation of autophagy. Mol Carcinog.

[90] Nowosad A, Besson A (2020). CDKN1B/p27 regulates autophagy via the control of Ragulator and MTOR activity in amino acid-deprived cells. Autophagy.

[91] Khattar E, Kumar V (2010). Mitogenic regulation of p271 gene is mediated by AP-1 transcription factors. J Biol Chem.

[92] Medema RH, Kops GJPL, Bos JL, Burgering BMT (2000). AFX-like Forkhead transcription factors mediate cell-cycle regulation by Ras and PKB through p27kip1. Nature.

[93] Brunet A, Bonni A, Zigmond MJ, Lin MZ, Juo P, Hu LS (1999). Akt promotes cell survival by phosphorylating and inhibiting a Forkhead transcription factor. Cell.

[94] Brunet A, Park J, Tran H, Hu LS, Hemmings BA, Greenberg ME (2001). Protein kinase SGK mediates survival signals by phosphorylating the forkhead transcription factor FKHRL1 (FOXO3a). Mol Cell Biol.

[95] Morishita D, Katayama R, Sekimizu K, Tsuruo T, Fujita N (2008). Pim kinases promote cell cycle progression by phosphorylating and down-regulating p27Kip1 at the transcriptional and posttranscriptional levels. Cancer Res.

[96] Wang C, Hou X, Mohapatra S, Ma Y, Cress WD, Pledger WJ (2005). Activation of p27Kip1 expression by E2F1. A negative feedback mechanism. J Biol Chem.

[97] Hao B, Zheng N, Schulman BA, Wu G, Miller JJ, Pagano M (2005). Structural basis of the Cks1-dependent recognition of p27Kip1 by the SCFSkp2 ubiquitin ligase. Mol Cell.

[98] Montagnoli A, Fiore F, Eytan E, Carrano AC, Draetta GF, Hershko A (1999). Ubiquitination of p27 is regulated by Cdk-dependent phosphorylation and trimeric complex formation. Genes Dev.

[99] Hattori T, Isobe T, Abe K, Kikuchi H, Kitagawa K, Oda T (2007). Pirh2 promotes ubiquitin-dependent degradation of the cyclin-dependent kinase inhibitor p27 *Kip1*. Cancer Res.

[100] Kamura T, Hara T, Matsumoto M, Ishida N, Okumura F, Hatakeyama S (2004). Cytoplasmic ubiquitin ligase KPC regulates proteolysis of p27Kip1 at G1 phase. Nat Cell Biol.

[101] Ishida N, Kitagawa M, Hatakeyama S, Nakayama KI (2000). Phosphorylation at serine 10, a major phosphorylation site of p27, increases its protein stability. J Bio Chem..

[102] Ishida N, Hara T, Kamura T, Yoshida M, Nakayama K, Nakayama KI (2002). Phosphorylation of p27 on serine 10 is required for its binding to CRM1 and nuclear export. J Biol Chem.

[103] Nacusi LP, Sheaff RJ (2006). Akt1 sequentially phosphorylates p27kip1 within a conserved but non-canonical region. Cell Div.

[104] Kajihara R, Fukushige S, Shioda N, Tanabe K, Fukunaga K, Inui S (2010). CaMKII phosphorylates serine 10 of p27 and confers apoptosis resistance to HeLa cells. Biochem Biophys Res Commun.

[105] Fujita N, Sato S, Katayama K, Tsuruo T (2002). Akt-dependent phosphorylation of p27Kip1Promotes Binding to 14-3-3 and cytoplasmic localization. J Biol Chem.

[106] Viglietto G, Motti ML, Bruni P, Melillo RM, D’Alessio A, Califano D (2002). Cytoplasmic relocalization and inhibition of the cyclin-dependent kinase inhibitor p27Kip1 by PKB/Akt-mediated phosphorylation in breast cancer. Nat Med.

[107] Tsvetkov LM, Yeh KH, Lee SJ, Sun H, Zhang H (1999). p27Kip1 ubiquitination and degradation is regulated by the SCFSkp2 complex through phosphorylated Thr187 in p27. Curr Biol.

[108] Carrano AC, Eytan E, Hershko A, Pagano M (1999). SKP2 is required for ubiquitin-mediated degradation of the CDK inhibitor p27. Nat Cell Biol.

[109] Sheaff RJ, Groudine M, Gordon M, Roberts JM, Clurman BE (1997). Cyclin E-CDK2 is a regulator of p27Kip1. Genes Dev.

[110] Osaki LH, Gama P (2013). MAPK signaling pathway regulates p27 phosphorylation at threonin 187 as part of the mechanism triggered by early-weaning to induce cell proliferation in rat gastric mucosa. PLoS ONE.

[111] Perez-Luna M, Aguasca M, Perearnau A, Serratosa J, Martinez-Balbas M, Jesus Pujol M (2012). PCAF regulates the stability of the transcriptional regulator and cyclin-dependent kinase inhibitor p27Kip1. Nucleic Acids Res.

[112] Payne SR, Kemp CJ (2003). p27(Kip1) (Cdkn1b)-deficient mice are susceptible to chemical carcinogenesis and may be a useful model for carcinogen screening. Toxicol Pathol.

[113] Philipp-Staheli J, Kim KH, Payne SR, Gurley KE, Liggitt D, Longton G (2002). Pathway-specific tumor suppression. Cancer Cell.

[114] Gao H, Ouyang X, Banach-Petrosky W, Borowsky AD, Lin Y, Kim M (2004). A critical role for *p27 *^*kip1*^ gene dosage in a mouse model of prostate carcinogenesis. Proc Natl Acad Sci.

[115] Sirma H, Broemel M, Stumm L, Tsourlakis T, Steurer S, Tennstedt P (2013). Loss of CDKN1B/p27Kip1 expression is associated with ERG fusion-negative prostate cancer, but is unrelated to patient prognosis. Oncol Lett.

[116] Dobashi Y, Tsubochi H, Minegishi K, Kitagawa M, Otani S, Ooi A (2017). Regulation of p27 by ubiquitin ligases and its pathological significance in human lung carcinomas. Hum Pathol.

[117] Loda M, Cukor B, Tam SW, Lavin P, Fiorentinc M, Draetta GF (1997). Increased proteasome-dependent degradation of the cyclin-dependent kinase inhibitor p27 in aggressive colorectal carcinomas. Nat Med.

[118] Zhu J, Li Y, Tian Z, Hua X, Gu J, Li J (2017). ATG7 overexpression is crucial for tumorigenic growth of bladder cancer in vitro and in vivo by targeting the ETS2/miRNA196b/FOXO1/p27 Axis. Mol Ther Nucleic Acids.

[119] Nycum LR, Smith LM, Farley JH, Kost ER, Method MW, Birrer MJ (2001). The role of p27 in Endometrial carcinoma. Gynecol Oncol.

[120] Jiao X, Wang B, Feng C, Song S, Tian B, Zhou C (2021). Formin-like protein 2 promotes cell proliferation by a p27-related mechanism in human breast cancer cells. BMC Cancer.

[121] Schiappacassi M, Lovat F, Canzonieri V, Belletti B, Berton S, Di Stefano D (2008). p27Kip1 expression inhibits glioblastoma growth, invasion, and tumor-induced neoangiogenesis. Mol Cancer Ther.

[122] Chen X, Cates JMM, Du YC, Jain A, Jung SY, Li XN (2020). Mislocalized cytoplasmic p27 activates PAK1-mediated metastasis and is a prognostic factor in osteosarcoma. Mol Oncol.

[123] Calvayrac O, Nowosad A, Cabantous S, Lin L, Figarol S, Jeannot P (2019). Cytoplasmic p27 ^Kip1^ promotes tumorigenesis via suppression of RhoB activity. J Pathol.

[124] Nagahara H, Vocero-Akbani AM, Snyder EL, Ho A, Latham DG, Lissy NA (1998). Transduction of full-length TAT fusion proteins into mammalian cells: TAT-p27Kip1 induces cell migration. Nat Med.

[125] Kruck S, Merseburger AS, Hennenlotter J, Scharpf M, Eyrich C, Amend B (2012). High cytoplasmic expression of p27 ^Kip1^ is associated with a worse cancer-specific survival in clear cell renal cell carcinoma. BJU Int.

[126] Chen G, Cheng Y, Zhang Z, Martinka M, Li G (2011). Prognostic significance of cytoplasmic p27 expression in human melanoma. Cancer Epidemiol Biomark Prev.

[127] Li Y, Nakka M, Kelly AJ, Lau CC, Krailo M, Barkauskas DA (2016). p27 Is a candidate prognostic biomarker and metastatic promoter in osteosarcoma. Cancer Res.

[128] Kouvaraki M, Gorgoulis VG, Rassidakis GZ, Liodis P, Markopoulos C, Gogas J (2002). High expression levels of p27 correlate with lymph node status in a subset of advanced invasive breast carcinomas. Cancer.

[129] Denicourt C, Saenz CC, Datnow B, Cui XS, Dowdy SF (2007). Relocalized p27Kip1 tumor suppressor functions as a cytoplasmic metastatic oncogene in melanoma. Cancer Res.

[130] Matsuoka S, Edwards MC, Bai C, Parker S, Zhang P, Baldini A (1995). p57KIP2, a structurally distinct member of the p21CIP1 Cdk inhibitor family, is a candidate tumor suppressor gene. Genes Dev.

[131] Creff J, Besson A (2020). Functional versatility of the CDK inhibitor p57Kip2. Front Cell Dev Biol.

[132] Watanabe H, Pan ZQ, Schreiber-Agus N, DePinho RA, Hurwitz J, Xiong Y (1998). Suppression of cell transformation by the cyclin-dependent kinase inhibitor p57 ^KIP2^ requires binding to proliferating cell nuclear antigen. Proc Natl Acad Sci.

[133] Zhang P, Liégeois NJ, Wong C, Finegold M, Hou H, Thompson JC (1997). Altered cell differentiation and proliferation in mice lacking p57KIP2 indicates a role in Beckwith-Wiedemann syndrome. Nature.

[134] Yan Y, Frisén J, Lee MH, Massagué J, Barbacid M (1997). Ablation of the CDK inhibitor p57Kip2 results in increased apoptosis and delayed differentiation during mouse development. Genes Dev.

[135] Yokoo T, Toyoshima H, Miura M, Wang Y, Iida KT, Suzuki H (2003). p57Kip2 regulates actin dynamics by binding and translocating LIM-kinase 1 to the nucleus. J Biol Chem.

[136] Yang N, Higuchi O, Ohashi K, Nagata K, Wada A, Kangawa K (1998). Cofilin phosphorylation by LIM-kinase 1 and its role in Rac-mediated actin reorganization. Nature.

[137] Vlachos P, Joseph B (2009). The Cdk inhibitor p57Kip2 contro LIM-kinase 1 activity and regulates actin cytoskeleton dynamics. Oncogene.

[138] Sakai K, Peraud A, Mainprize T, Nakayama J, Tsugu A, Hongo K (2004). Inducible expression of p57 ^KIP2^ inhibits glioma cell motility and invasion. J Neurooncol.

[139] Joaquin M, Gubern A, González-Nuñez D, Josué Ruiz E, Ferreiro I, de Nadal E (2012). The p57 CDKi integrates stress signals into cell-cycle progression to promote cell survival upon stress. EMBO J.

[140] Chang TS, Kim MJ, Ryoo K, Park J, Eom SJ, Shim J (2003). p57KIP2 modulates stress-activated signaling by inhibiting c-jun NH2-terminal kinase/stress-activated protein kinase. J Biol Chem.

[141] Vlachos P, Nyman U, Hajji N, Joseph B (2007). The cell cycle inhibitor p57Kip2 promotes cell death via the mitochondrial apoptotic pathway. Cell Death Differ.

[142] Gonzalez S, Perez-Perez MM, Hernando E, Serrano M, Cordon-Cardo C (2005). p73β-mediated apoptosis requires p57kip2 induction and IEX-1 inhibition. Cancer Res.

[143] Reynaud EG, Leibovitch MP, Tintignac LAJ, Pelpel K, Guillier M, Leibovitch SA (2000). Stabilization of MyoD by direct binding to p57Kip2. J Biol Chem.

[144] Joseph B, Wallén-Mackenzie Å, Benoit G, Murata T, Joodmardi E, Okret S (2003). p57 ^Kip2^ cooperates with Nurr1 in developing dopamine cells. Proc Natl Acad Sci.

[145] Joseph B, Andersson ER, Vlachos P, Södersten E, Liu L, Teixeira AI (2009). p57Kip2 is a repressor of Mash1 activity and neuronal differentiation in neural stem cells. Cell Death Differ.

[146] Ma Y, Cress WD (2007). Transcriptional upregulation of p57 (Kip2) by the cyclin-dependent kinase inhibitor BMS-387032 is E2F dependent and serves as a negative feedback loop limiting cytotoxicity. Oncogene.

[147] Cucciolla V, Borriello A, Criscuolo M, Sinisi AA, Bencivenga D, Tramontano A (2007). Histone deacetylase inhibitors upregulate p57Kip2 level by enhancing its expression through Sp1 transcription factor. Carcinogenesis.

[148] Schipani E, Ryan HE, Didrickson S, Kobayashi T, Knight M, Johnson RS (2001). Hypoxia in cartilage: HIF-1α is essential for chondrocyte growth arrest and survival. Genes Dev.

[149] Scandura JM, Boccuni P, Massagué J, Nimer SD (2004). Transforming growth factor β-induced cell cycle arrest of human hematopoietic cells requires p57KIP2 up-regulation. Proc Natl Acad Sci.

[150] Vaccarello G, Figliola R, Cramerotti S, Novelli F, Maione R (2006). p57Kip2 is induced by MyoD through a p73-dependent pathway. J Mol Biol.

[151] Guo H, Jing L, Cheng Y, Atsaves V, Lv Y, Wu T (2016). Down-regulation of the cyclin-dependent kinase inhibitor p57 is mediated by Jab1/Csn5 in hepatocarcinogenesis. Hepatology.

[152] Giovannini C, Gramantieri L, Minguzzi M, Fornari F, Chieco P, Grazi GL (2012). CDKN1C/P57 is regulated by the notch target gene Hes1 and induces senescence in human hepatocellular carcinoma. Am J Pathol.

[153] Kikuchi T, Toyota M, Itoh F, Suzuki H, Obata T, Yamamoto H (2002). Inactivation of p57KIP2 by regional promoter hypermethylation and histone deacetylation in human tumors. Oncogene.

[154] Yamaguchi T, Cubizolles F, Zhang Y, Reichert N, Kohler H, Seiser C (2010). Histone deacetylases 1 and 2 act in concert to promote the G1-to-S progression. Genes Dev.

[155] Fan T, Hagan JP, Kozlov SV, Stewart CL, Muegge K (2005). Lsh controls silencing of the imprinted *Cdkn1c* gene. Development.

[156] Guo J, Cai J, Yu L, Tang H, Chen C, Wang Z (2011). EZH2 regulates expression of p57 and contributes to progression of ovarian cancer *in vitro* and *in vivo*. Cancer Sci.

[157] Naito M, Mori M, Inagawa M, Miyata K, Hashimoto N, Tanaka S (2016). Dnmt3a regulates proliferation of muscle satellite cells via p57Kip2. PLoS Genet.

[158] Kamura T, Hara T, Kotoshiba S, Yada M, Ishida N, Imaki H (2003). Degradation of p57 ^*Kip2*^ mediated by SCF ^Skp2^ -dependent ubiquitylation. Proc Natl Acad Sci.

[159] Kim M, Nakamoto T, Nishimori S, Tanaka K, Chiba T (2008). A new ubiquitin ligase involved in p57 ^KIP2^ proteolysis regulates osteoblast cell differentiation. EMBO Rep.

[160] Lin M, Zhang J, Bouamar H, Wang Z, Sun LZ, Zhu X (2022). Fbxo22 promotes cervical cancer progression via targeting p57Kip2 for ubiquitination and degradation. Cell Death Dis.

[161] Zhang L, Chen J, Ning D, Liu Q, Wang C, Zhang Z (2019). FBXO22 promotes the development of hepatocellular carcinoma by regulating the ubiquitination and degradation of p21. J Exp Clin Cancer Res.

[162] Yi L, Wang H, Li W, Ye K, Xiong W, Yu H (2021). The FOXM1/RNF26/p57 axis regulates the cell cycle to promote the aggressiveness of bladder cancer. Cell Death Dis.

[163] Zhao R, Yang HY, Shin J, Phan L, Fang L, Che TF (2013). CDK inhibitor p57 ^Kip2^ is downregulated by Akt during HER2-mediated tumorigenicity. Cell Cycle.

[164] Kettenbach AN, Schweppe DK, Faherty BK, Pechenick D, Pletnev AA, Gerber SA (2011). Quantitative phosphoproteomics identifies substrates and functional modules of aurora and polo-like kinase activities in mitotic cells. Sci Signal..

[165] Mishra S, Lin CL, Huang THM, Bouamar H, Sun LZ (2014). MicroRNA-21 inhibits p57Kip2 expression in prostate cancer. Mol Cancer.

[166] Sun K, Wang W, Zeng JJ, Wu CT, Lei ST, Li GX (2011). MicroRNA-221 inhibits CDKN1C/p57 expression in human colorectal carcinoma. Acta Pharmacol Sin.

[167] Ito Y, Takeda T, Wakasa KI, Tsujimoto M, Matsuura N (2001). Expression of p57/Kip2 protein in pancreatic adenocarcinoma. Pancreas.

[168] Biaoxue R, Xiguang C, Hua L, Hui M, Shuanying Y, Wei Z (2011). Decreased expression of decorin and p57(KIP2) correlates with poor survival and lymphatic metastasis in lung cancer patients. Int J Biol Markers.

[169] Yang C, Nan H, Ma J, Jiang L, Guo Q, Han L (2015). High Skp2/Low p57 ^Kip2^ expression is associated with poor prognosis in human breast carcinoma. Breast Cancer.

[170] Oya M, Schulz WA (2000). Decreased expression of p57KIP2 mRNA in human bladder cancer. Br J Cancer.

[171] Qiu Z, Li Y, Zeng B, Guan X, Li H (2018). Downregulated CDKN1C/p57kip2 drives tumorigenesis and associates with poor overall survival in breast cancer. Biochem Biophys Res Commun.

[172] Kavanagh E, Joseph B (2011). The hallmarks of CDKN1C (p57, KIP2) in cancer. Biochimica et Biophysica Acta Rev Cancer..

[173] Weis B, Schmidt J, Maamar H, Raj A, Lin H, Tóth C (2015). Inhibition of intestinal tumor formation by deletion of the DNA methyltransferase 3a. Oncogene.

[174] Ito J, Yamagata K, Shinohara H, Shima Y, Katsumoto T, Aikawa Y (2023). Dual inhibition of EZH1/2 induces cell cycle arrest of B cell acute lymphoblastic leukemia cells through upregulation of CDKN1C and TP53INP1. Int J Hematol.

[175] Lin W, Wang K, Mo J, Wang L, Song Z, Jiang H (2023). <scp>PIK3R3</scp> is upregulated in liver cancer and activates Akt signaling to control cancer growth by regulation of <scp>CDKN1C</scp> and <scp>SMC1A</scp>. Cancer Med.

[176] Oka T, Higa T, Sugahara O, Koga D, Nakayama S, Nakayama KI (2023). Ablation of p57+ quiescent cancer stem cells suppresses recurrence after chemotherapy of intestinal tumors. Cancer Res.

[177] Cánepa ET, Scassa ME, Ceruti JM, Marazita MC, Carcagno AL, Sirkin PF, et al. INK4 proteins, a family of mammalian CDK inhibitors with novel biological functions. IUBMB Life. 2007;59(7):419–26. https://iubmb.onlinelibrary.wiley.com/doi/abs/10.1080/15216540701488358 10.1080/15216540701488358 17654117

[178] Serrano M, Hannon GJ, Beach D (1993). A new regulatory motif in cell-cycle control causing specific inhibition of cyclin D/CDK4. Nature.

[179] Hannon GJ, Beach D (1994). pl5INK4B is a potentia| effector of TGF-β-induced cell cycle arrest. Nature.

[180] Forget A, Ayrault O, Den Besten W, Kuo ML, Sherr CJ, Roussel MF (2008). Differential post-transcriptional regulation of two Ink4 proteins, p18 Ink4c and p19Ink4d. Cell Cycle.

[181] Suzuki H, Zhou X, Yin J, Lei J, Jiang HY, Suzuki Y, et al. Intragenic mutations of CDKN2B and CDKN2A in primary human esophageal cancers. Vol. 4, Human Molecular Genetics. 1995. 10.1093/hmg/4.10.1883 8595411

[182] Krimpenfort P, Quon KC, Mooi WJ, Loonstra A, Berns A (2001). Loss of p16Ink4a confers susceptibility to metastatic melanoma in mice. Nature.

[183] Hirai H, Roussel MF, Kato JY, Ashmun RA, Sherr CJ (1995). Novel INK4 Proteins, p19 and p18, are specific inhibitors of the cyclin D-dependent kinases CDK4 and CDK6. Mol Cell Biol.

[184] Venkataramani R, Swaminathan K, Marmorstein R (1998). Crystal structure of the CDK4/6 inhibitory protein p18INK4c provides insights into ankyrin-like repeat structure/function and tumor-derived p16INK4 mutations. Nat Struct Biol.

[185] Kumar A, Balbach J, Kumar A, Balbach J, Uversky N (2021). Folding and stability of ankyrin repeats control biological protein function. Biomolecules.

[186] Jeffrey PD, Tong L, Pavletich NP (2000). Structural basis of inhibition of CDK–cyclin complexes by INK4 inhibitors. Genes Dev.

[187] Sherr CJ, Roberts JM (1999). CDK inhibitors: positive and negative regulators of G1-phase progression. Genes Dev.

[188] Schirripa A, Sexl V, Kollmann K (2022). Cyclin-dependent kinase inhibitors in malignant hematopoiesis. Front Oncol.

[189] Ohtani N, Zebedee Z, Huot TJG, Stinson JA, Sugimoto M, Ohashi Y (2001). Opposing effects of Ets and Id proteins on p16INK4a expression during cellular senescence. Nature.

[190] Li J, Poi MJ, Tsai MD (2011). Regulatory mechanisms of tumor suppressor P16INK4A and their relevance to cancer. Biochemistry.

[191] Russo AA, Tong L, Lee JO, Jeffrey PD, Pavletich NP (1998). Structural basis for inhibition of the cyclin-dependent kinase Cdk6 by the tumour suppressor p16(INK4a). Nature.

[192] Reynisdóttir I, Polyak K, Iavarone A, Massagué J (1995). Kip/Cip and Ink4 Cdk inhibitors cooperate to induce cell cycle arrest in response to TGF-beta. Genes Dev.

[193] Bu YC, Hong SC, Ko K, Cho YY, Zhu F, Bong SK (2005). The tumor suppressor p16INK4a prevents cell transformation through inhibition of c-Jun phosphorylation and AP-1 activity. Nat Struct Mol Biol.

[194] Souza-Rodrígues E, Estanyol JM, Friedrich-Heineken E, Olmedo E, Vera J, Canela N (2007). Proteomic analysis of p16ink4a-binding proteins. Proteomics.

[195] Krishnamurthy J, Torrice C, Ramsey MR, Kovalev GI, Al-Regaiey K, Su L (2004). Ink4a/Arf expression is a biomarker of aging. J Clin Investigat..

[196] Dietrich N, Bracken AP, Trinh E, Schjerling CK, Koseki H, Rappsilber J (2007). Bypass of senescence by the polycomb group protein CBX8 through direct binding to the INK4A-ARF locus. EMBO J.

[197] Zhu J, Woods D, McMahon M, Bishop JM (1998). Senescence of human fibroblasts induced by oncogenic Raf. Genes Dev.

[198] Lin AW, Barradas M, Stone JC, van Aelst L, Serrano M, Lowe SW (1998). Premature senescence involving p53 and p16 is activated in response to constitutive MEK/MAPK mitogenic signaling. Genes Dev.

[199] Gonzalez S, Serrano M (2006). a new mechanism of inactivation of the INK4/ARF locus. Cell Cycle.

[200] Wang X, Pan L, Feng Y, Wang Y, Han Q, Han L (2008). p300 plays a role in p16INK4a expression and cell cycle arrest. Oncogene.

[201] Wang X, Feng Y, Xu L, Chen Y, Zhang Y, Su D (2008). YY1 restrained cell senescence through repressing the transcription of p16. Biochimica et Biophysica Acta Mol Cell Res..

[202] Li Y, Nichols MA, Shay JW, Xiong Y (1994). Transcriptional repression of the D-type cyclin-dependent kinase inhibitor p16 by the retinoblastoma susceptibility gene product pRb. Cancer Res.

[203] Gil J, Bernard D, Martínez D, Beach D (2004). Polycomb CBX7 has a unifying role in cellular lifespan. Nat Cell Biol.

[204] Bracken AP, Kleine-Kohlbrecher D, Dietrich N, Pasini D, Gargiulo G, Beekman C (2007). The Polycomb group proteins bind throughout the *INK4A-ARF* locus and are disassociated in senescent cells. Genes Dev.

[205] Frescas D, Guardavaccaro D, Bassermann F, Koyama-Nasu R, Pagano M (2007). JHDM1B/FBXL10 is a nucleolar protein that represses transcription of ribosomal RNA genes. Nature.

[206] Tzatsos A, Pfau R, Kampranis SC, Tsichlis PN (2009). Ndy1/KDM2B immortalizes mouse embryonic fibroblasts by repressing the *Ink4a* / *Arf* locus. Proc Natl Acad Sci.

[207] Nuovo GJ, Plaia TW, Belinsky SA, Baylin SB, Herman JG (1999). *In situ* detection of the hypermethylation-induced inactivation of the *p16* gene as an early event in oncogenesis. Proc Natl Acad Sci.

[208] Bai J, Zhang X, Liu B, Wang H, Du Z, Song J (2017). Silencing DNA methyltransferase 1 leads to the activation of the esophageal suppressor gene p16 in vitro and in vivo. Oncol Lett.

[209] Lu R, Wang X, Chen ZF, Sun DF, Tian XQ, Fang JY (2007). Inhibition of the extracellular signal-regulated kinase/mitogen-activated protein kinase pathway decreases DNA methylation in colon cancer cells. J Biol Chem.

[210] Serrano M, Lin AW, McCurrach ME, Beach D, Lowe SW (1997). Oncogenic ras provokes premature cell senescence associated with accumulation of p53 and p16INK4a. Cell.

[211] Agger K, Cloos PAC, Rudkjær L, Williams K, Andersen G, Christensen J (2009). The H3K27me3 demethylase JMJD3 contributes to the activation of the *INK4A–ARF* locus in response to oncogene- and stress-induced senescence. Genes Dev.

[212] Al-Khalaf HH, Hendrayani SF, Aboussekhra A (2011). The Atr protein kinase controls UV-dependent upregulation of p16INK4A through inhibition of Skp2-related polyubiquitination/degradation. Mol Cancer Res.

[213] Gump J, Stokoe D, McCormick F (2003). Phosphorylation of p16 correlates with Cdk4 association. J Biol Chem.

[214] Guo Y, Yuan C, Weghorst CM, Li J (2010). IKKβ specifically binds to P16 and phosphorylates Ser8 of P16. Biochem Biophys Res Commun.

[215] Wang X, Huang Y, Zhao J, Zhang Y, Lu J, Huang B (2012). Suppression of PRMT6-mediated arginine methylation of p16 protein potentiates its ability to arrest A549 cell proliferation. Int J Biochem Cell Biol.

[216] Chen P, Zindy F, Abdala C, Liu F, Li X, Roussel MF (2003). Progressive hearing loss in mice lacking the cyclin-dependent kinase inhibitor lnk4d. Nat Cell Biol.

[217] Latres E, Malumbres M, Sotillo R, Martín J, Ortega S, Martín-Caballero J (2000). Limited overlapping roles of p15(INK4b) and p18(INK4c) cell cycle inhibitors in proliferation and tumorigenesis. EMBO J.

[218] Krimpenfort P, Ijpenberg A, Song JY, Van Der Valk M, Nawijn M, Zevenhoven J (2007). p15Ink4b is a critical tumour suppressor in the absence of p16Ink4a. Nature.

[219] Hussussian CJ, Struewing JP, Goldstein AM, Higgins PAT, Ally DS, Sheahan MD (1994). Germline p16 mutations in familial melanoma. Nat Genet.

[220] Foulkes WD, Flanders TY, Pollock PM, Hayward NK (1997). The CDKN2A (p16) gene and human cancer. Mol Med.

[221] Sweeney SM, Cerami E, Baras A, Pugh TJ, Schultz N, Stricker T (2017). AACR project genie: powering precision medicine through an international consortium. Cancer Discov.

[222] Brenner AJ, Aldaz CM (1995). Chromosome 9p allelic loss and pl6/CDKN2 in breast cancer and evidence of pl6 inactivation in immortal breast epithelial cells. Cancer Res.

[223] Nobori S, Carson DA, Sauter ER, Shafarenko M, Mitsunaga S, Ridge JA (1994). Higher frequency of alterations in the pl6/CDKN2 gene in squamous cell carcinoma cell lines than in primary tumors of the head and neck. Cancer Res.

[224] Igaki H, Sasaki H, Tachimori Y, Kato H, Watanabe H, Kimura T (1995). Mutation frequency of the p16/CDKN2 gene in primary cancers in the upper digestive tract. Cancer Res.

[225] Simboeck E, Ribeiro JD, Teichmann S, Di Croce L (2011). Epigenetics and senescence: learning from the INK4-ARF locus. Biochem Pharmacol.

[226] Narita M, Nũnez S, Heard E, Narita M, Lin AW, Hearn SA (2003). Rb-mediated heterochromatin formation and silencing of E2F target genes during cellular senescence. Cell.

[227] Foos G, García-Ramírez JJ, Galang CK, Hauser CA (1998). Elevated expression of Ets2 or distinct portions of Ets2 can reverse Ras-mediated cellular transformation. J Bio Chem..

[228] Takahashi A, Ohtani N, Yamakoshi K, Iida SI, Tahara H, Nakayama K (2006). Mitogenic signalling and the p16INK4a-Rb pathway cooperate to enforce irreversible cellular senescence. Nat Cell Biol.

[229] Herman JG, Civin CI, Issa JPJ, Collector MI, Sharkis SJ, Baylin SB (1997). Distinct patterns of inactivation of p15(INK4B) and p16(INK4A) characterize the major types of hematological malignancies. Cancer Res.

[230] Jha AK, Nikbakht M, Jain V, Capalash N, Kaur J (2012). P16 ink4a and p15 ink4b gene promoter methylation in cervical cancer patients. Oncol Lett.

[231] Viswanathan M, Tsuchida N, Shanmugam G (2003). Promoter hypermethylation profile of tumor-associated genes p16, p15, hMLH1, MGMT and E-cadherin in oral squamous cell carcinoma. Int J Cancer.

[232] Moselhy SS, Kumosani TA, Kamal I, Jalal J, Jabaar HSA, Dalol A (2015). Hypermethylation of P15, P16, and E-cadherin genes in ovarian cancer. Toxicol Ind Health.

[233] Ng MHL, Chung YF, Lo KW, Wickham NWR, Lee JCK, Huang DP (1997). Frequent hypermethylation of p16 and p15 genes in multiple myeloma. Blood.

[234] Inoue KA, Fry E (2018). Aberrant expression of p16INK4a in human cancers – a new biomarker?. Cancer Rep Rev..

[235] Kim BN, Yamamoto H, Ikeda K, Damdinsuren B, Sugita Y, Ngan CY (2005). Methylation and expression of p16INK4 tumor suppressor gene in primary colorectal cancer tissues. Int J Oncol.

[236] King-Yin Lam A, Ong K, Ho YH (2006). Colorectal mucinous adenocarcinoma: The clinicopathologic features and significance of p16 and p53 expression. Dis Colon Rectum.

[237] J. S, N. M. Promitotic and cyclin-dependent kinase inhibitor proteins show significant correlation with distant metastasis in breast cancer patients. Laboratory Investigation. 2018;98(Supplement 1).

[238] Chang PH, Wang HM, Kuo YC, Lee LY, Liao CJ, Kuo HC (2021). Circulating p16-positive and p16-negative tumor cells serve as independent prognostic indicators of survival in patients with head and neck squamous cell carcinomas. J Pers Med..

[239] Farooq U, Notani D (2022). Transcriptional regulation of INK4/ARF locus by cis and trans mechanisms. Front Cell Dev Biol.

[240] Chen CR, Kang Y, Siegel PM, Massagué J (2002). E2F4/5 and p107 as smad cofactors linking the TGFΒ receptor to c-myc repression. Cell.

[241] Reynisdóttir I, Polyak K, Iavarone A, Massagué J (1995). Kip/Cip and Ink4 Cdk inhibitors cooperate to induce cell cycle arrest in response to TGF-β. Genes Dev.

[242] Xia Y, Liu Y, Yang C, Simeone DM, Sun TT, DeGraff DJ (2021). Dominant role of CDKN2B/p15INK4B of 9p213 tumor suppressor hub in inhibition of cell-cycle and glycolysis. Nat Commun.

[243] Kheradmand Kia S, Solaimani Kartalaei P, Farahbakhshian E, Pourfarzad F, von Lindern M, Verrijzer CP (2009). EZH2-dependent chromatin looping controls INK4a and INK4b, but not ARF, during human progenitor cell differentiation and cellular senescence. Epigenetics Chromatin.

[244] Jacobs JJL, Kieboom K, Marino S, DePinho RA, van Lohuizen M (1999). The oncogene and Polycomb-group gene bmi-1 regulates cell proliferation and senescence through the ink4a locus. Nature.

[245] Kotake Y, Nakagawa T, Kitagawa K, Suzuki S, Liu N, Kitagawa M (2011). Long non-coding RNA ANRIL is required for the PRC2 recruitment to and silencing of p15INK4B tumor suppressor gene. Oncogene.

[246] Rajaraman P, Melin BS, Wang Z, McKean-Cowdin R, Michaud DS, Wang SS (2012). Genome-wide association study of glioma and meta-analysis. Hum Genet.

[247] Turnbull C, Ahmed S, Morrison J, Pernet D, Renwick A, Maranian M (2010). Genome-wide association study identifies five new breast cancer susceptibility loci. Nat Genet.

[248] Pasmant E, Laurendeau I, Héron D, Vidaud M, Vidaud D, Bièche I (2007). Characterization of a germ-line deletion, including the entire INK4/ARF locus, in a melanoma-neural system tumor family: identification of ANRIL, an antisense noncoding RNA whose expression coclusters with ARF. Cancer Res.

[249] Qiu JJ, Wang Y, Liu YL, Zhang Y, Ding JX, Hua KQ (2016). The long non-coding RNA ANRIL promotes proliferation and cell cycle progression and inhibits apoptosis and senescence in epithelial ovarian cancer. Oncotarget.

[250] Hitomi T, Matsuzaki Y, Yasuda S, Kawanaka M, Yogosawa S, Koyama M (2007). is involved in the transcriptional repression of the p15 ^INK4b^ gene. FEBS Lett.

[251] Ogura T, Azuma K, Sato J, Kinowaki K, Takayama KI, Takeiwa T (2021). OCT1 is a poor prognostic factor for breast cancer patients and promotes cell proliferation via inducing NCAPH. Int J Mol Sci.

[252] Zhong Y, Huang H, Chen M, Huang J, Wu Q, Yan GR (2017). POU2F1 over-expression correlates with poor prognoses and promotes cell growth and epithelial-to-mesenchymal transition in hepatocellular carcinoma. Oncotarget.

[253] Wang YP, Song GH, Chen J, Xiao C, Li C, Zhong L (2016). Elevated OCT1 participates in colon tumorigenesis and independently predicts poor prognoses of colorectal cancer patients. Tumor Bio..

[254] Li JM, Datto MB, Shen X, Hu PP, Yu Y, Wang XF (1998). , but not Sp3, functions to mediate promoter activation by TGF-beta through canonical Sp1 binding sites. Nucleic Acids Res.

[255] Sandhu C, Garbe J, Bhattacharya N, Daksis J, Pan CH, Yaswen P (1997). Transforming growth factor β stabilizes p15 ^*INK4B*^ protein, increases p15 ^*INK4B*^ -cdk4 complexes, and inhibits Cyclin D1-cdk4 association in human mammary epithelial cells. Mol Cell Biol.

[256] Katayama K, Nakamura A, Sugimoto Y, Tsuruo T, Fujita N (2008). FOXO transcription factor-dependent p15INK4b and p19INK4d expression. Oncogene.

[257] Ballif BA, Villén J, Beausoleil SA, Schwartz D, Gygi SP (2004). Phosphoproteomic analysis of the developing mouse brain. Mol Cell Proteomics.

[258] Gil J, Peters G (2006). Regulation of the INK4b–ARF–INK4a tumour suppressor locus: all for one or one for all. Nat Rev Mol Cell Biol.

[259] Schuster K, Venkateswaran N, Rabellino A, Girard L, Peña-Llopis S, Scaglioni PP. Nullifying the *CDKN2AB* Locus Promotes Mutant K-ras Lung Tumorigenesis. Mol Cancer Res. 2014;12(6):912–23. 10.1158/1541-7786.MCR-13-0620-T PMC4058359 24618618

[260] Tu Q, Hao J, Zhou X, Yan L, Dai H, Sun B (2018). CDKN2B deletion is essential for pancreatic cancer development instead of unmeaningful co-deletion due to juxtaposition to CDKN2A. Oncogene.

[261] Inoue K, Fry EA (2018). Aberrant expression of p16INK4a in human cancers - a new biomarker?. Cancer Rep Rev..

[262] Park SS, Lee YK, Park SH, Lim SB, Choi YW, Shin JS (2023). p15INK4B is an alternative marker of senescent tumor cells in colorectal cancer. Heliyon..

[263] Coppé JP, Desprez PY, Krtolica A, Campisi J (2010). The senescence-associated secretory phenotype: the dark side of tumor suppression. Annu Rev Pathol.

[264] Gorgoulis V, Adams PD, Alimonti A, Bennett DC, Bischof O, Bishop C (2019). Cellular senescence: defining a path forward. Cell.

[265] Takasugi M, Yoshida Y, Hara E, Ohtani N (2023). The role of cellular senescence and SASP in tumour microenvironment. FEBS J.

[266] Xiao S, Qin D, Hou X, Tian L, Yu Y, Zhang R (2023). Cellular senescence: a double-edged sword in cancer therapy. Front Oncol.

[267] Buj R, Leon KE, Anguelov MA, Aird KM (2021). Suppression of p16 alleviates the senescence-associated secretory phenotype. Aging.

[268] Blais A, Labrie Y, Pouliot F, Lachance Y, Labrie C (1998). Structure of the gene encoding the human cyclin-dependent kinase inhibitor p18 and Mutational Analysis In Breast Cancer. Biochem Biophys Res Commun.

[269] Latres E (2000). Limited overlapping roles of P15INK4b and P18INK4c cell cycle inhibitors in proliferation and tumorigenesis. EMBO J.

[270] Franklin DS, Godfrey VL, Lee H, Kovalev GI, Schoonhoven R, Chen-Kiang S (1998). CDK inhibitors p18(INK4c) and p27(Kip1) mediate two separate pathways to collaboratively suppress pituitary tumorigenesis. Genes Dev.

[271] Bai F, Pei XH, Godfrey VL, Xiong Y (2003). Haploinsufficiency of p18(INK4c) sensitizes mice to carcinogen-induced tumorigenesis. Mol Cell Biol.

[272] Bai F, Pei XH, Pandolfi PP, Xiong Y (2006). p18 Ink4c and Pten constrain a positive regulatory loop between cell growth and cell cycle control. Mol Cell Biol.

[273] Zindy F, Nilsson LM, Nguyen L, Meunier C, Smeyne RJ, Rehg JE (2003). Hemangiosarcomas, medulloblastomas, and other tumors in Ink4c/p53-null mice. Cancer Res.

[274] Blais A, Monté D, Pouliot F, Labrie C (2002). Regulation of the human cyclin-dependent kinase inhibitor p18 by the transcription factors E2F1 and Sp1. J Biol Chem.

[275] Sánchez-Aguilera A, Delgado J, Camacho FI, Sánchez-Beato M, Sánchez L, Montalbán C (2004). Silencing of the p18INK4c gene by promoter hypermethylation in Reed-Sternberg cells in Hodgkin lymphomas. Blood.

[276] Cui H, Zhao C, Gong P, Wang L, Wu H, Zhang K (2015). DNA methyltransferase 3A promotes cell proliferation by silencing CDK inhibitor p18INK4C in gastric carcinogenesis. Sci Rep.

[277] Zhou M, Mao Y, Yu S, Li Y, Yin R, Zhang Q (2020). LINC00673 represses CDKN2C and promotes the proliferation of esophageal squamous cell carcinoma cells by EZH2-mediated H3K27 trimethylation. Front Oncol.

[278] Chen H, Su Y, Yang L, Xi L, Li X, Lan B (2023). CBX8 promotes lung adenocarcinoma growth and metastasis through transcriptional repression of CDKN2C and SCEL. J Cell Physiol.

[279] Tang J, Meng Q, Shi R, Xu Y (2020). PRMT6 serves an oncogenic role in lung adenocarcinoma via regulating p18. Mol Med Rep.

[280] Forget A, Ayrault O, den Besten W, Kuo ML, Sherr CJ, Roussel MF (2008). Differential post-transcriptional regulation of two Ink4 proteins, p18 Ink4c and p19 Ink4d. Cell Cycle.

[281] Li Y, Shi F, Hu J, Xie L, Zhao L, Tang M (2021). Stabilization of p18 by deubiquitylase CYLD is pivotal for cell cycle progression and viral replication. NPJ Precis Oncol.

[282] Uziel T, Zindy F, Sherr CJ, Roussel MF (2006). The CDK inhibitor p18Ink4c is a tumor suppressor in medulloblastoma. Cell Cycle.

[283] Morishita A, Masaki T, Yoshiji H, Nakai S, Ogi T, Miyauchi Y (2004). Reduced expression of cell cycle regulator p18INK4C in human hepatocellular carcinoma. Hepatology.

[284] Bartkova J, Thullberg M, Rajpert-De Meyts E, Skakkebæk NE, Bartek J (2000). Cell cycle regulators in testicular cancer: loss of p18(INK4) marks progression from carcinoma in situ to invasive germ cell tumours. Int J Cancer.

[285] Van Veelen W, Van Gasteren CJR, Acton DS, Franklin DS, Berger R, Lips CJM (2008). Synergistic effect of oncogenic RET and loss of p18 on medullary thyroid carcinoma development. Cancer Res.

[286] Van Veelen W, Klompmaker R, Gloerich M, Van Gasteren CJR, Kalkhoven E, Berger R (2009). P18 is a tumor suppressor gene involved in human medullary thyroid carcinoma and pheochromocytoma development. Int J Cancer.

[287] Cui H, Zhao C, Gong P, Wang L, Wu H, Zhang K (2015). DNA methyltransferase 3A promotes cell proliferation by silencing CDK inhibitor p18 INK4C in gastric carcinogenesis. Sci Rep.

[288] Li Q, Jiang B, Guo J, Shao H, Del Priore IS, Chang Q (2022). INK4 tumor suppressor proteins mediate resistance to CDK4/6 kinase inhibitors. Cancer Discov.

[289] Schmalzbauer BS, Thondanpallil T, Heller G, Schirripa A, Sperl CM, Mayer IM (2022). CDK6 degradation is counteracted by p16INK4A and p18INK4C in AML. Cancers.

[290] Zindy F, Cunningham JJ, Sherr CJ, Jogal S, Smeyne RJ, Roussel MF (1999). Postnatal neuronal proliferation in mice lacking Ink4d and Kip1 inhibitors of cyclin-dependent kinases. Proc Natl Acad Sci.

[291] Zindy F, den Besten W, Chen B, Rehg JE, Latres E, Barbacid M (2001). Control of spermatogenesis in mice by the cyclin D-dependent kinase inhibitors p18 ^Ink4c^ and p19 ^Ink4d^. Mol Cell Biol.

[292] Han X, Zhang J, Peng Y, Peng M, Chen X, Chen H (2017). Unexpected role for p19INK4d in posttranscriptional regulation of GATA1 and modulation of human terminal erythropoiesis. Blood.

[293] Sonzogni SV, Ogara MF, Belluscio LM, Castillo DS, Scassa ME, Cánepa ET (2014). p19INK4d is involved in the cellular senescence mechanism contributing to heterochromatin formation. Biochimica et Biophysica Acta Gene Sub..

[294] Sonzogni SV, Ogara MF, Castillo DS, Sirkin PF, Radicella JP, Cánepa ET (2015). Nuclear translocation of p19INK4d in response to oxidative DNA damage promotes chromatin relaxation. Mol Cell Biochem.

[295] Ceruti JM, Scassa ME, Fló JM, Varone CL, Cánepa ET (2005). Induction of p19INK4d in response to ultraviolet light improves DNA repair and confers resistance to apoptosis in neuroblastoma cells. Oncogene.

[296] Scassa ME, Marazita MC, Ceruti JM, Carcagno AL, Sirkin PF, González-Cid M (2007). Cell cycle inhibitor, p19INK4d, promotes cell survival and decreases chromosomal aberrations after genotoxic insult due to enhanced DNA repair. DNA Repair.

[297] Carcagno AL, Marazita MC, Ogara MF, Ceruti JM, Sonzogni SV, Scassa ME (2011). E2F1-mediated upregulation of p19INK4d determines its periodic expression during cell cycle and regulates cellular proliferation. PLoS ONE.

[298] Carcagno AL, Giono LE, Marazita MC, Castillo DS, Pregi N, Cánepa ET (2012). E2F1 induces p19INK4d, a protein involved in the DNA damage response, following UV irradiation. Mol Cell Biochem.

[299] Tavera-Mendoza L, Wang TT, Lallemant B, Zhang R, Nagai Y, Bourdeau V (2006). Convergence of vitamin D and retinoic acid signalling at a common hormone response element. EMBO Rep.

[300] Korf K, Wodrich H, Haschke A, Ocampo C, Harder L, Gieseke F (2014). The PML domain of PML–RARα blocks senescence to promote leukemia. Proc Natl Acad Sci.

[301] Zhou H, Cai Y, Liu D, Li M, Sha Y, Zhang W (2018). Pharmacological or transcriptional inhibition of both HDAC 1 and 2 leads to cell cycle blockage and apoptosis via p21 Waf1/Cip1 and p19 INK4d upregulation in hepatocellular carcinoma. Cell Prolif.

[302] Thullberg M, Bartek J, Lukas J (2000). Ubiquitin/proteasome-mediated degradation of p19INK4d determines its periodic expression during the cell cycle. Oncogene.

[303] Forget A, Ayrault O, den Besten W, Kuo ML, Sherr CJ, Roussel MF (2008). Differential post-transcriptional regulation of two Ink4 proteins, p18Ink4c and p19Ink4d. Cell Cycle.

[304] Kumar A, Gopalswamy M, Wolf A, Brockwell DJ, Hatzfeld M, Balbach J (2018). Phosphorylation-induced unfolding regulates p19 ^INK4d^ during the human cell cycle. Proc Natl Acad Sci.

[305] Penas C, Ramachandran V, Ayad NG (2012). The APC/C ubiquitin ligase: from cell biology to tumorigenesis. Front Oncol.

[306] Msallam M, Sun H, Meledin R, Franz P, Brik A (2020). Examining the role of phosphorylation of p19 ^INK4d^ in its stability and ubiquitination using chemical protein synthesis. Chem Sci.

[307] Marazita MC, Ogara MF, Sonzogni SV, Martí M, DUSETTI NJ, Pignataro OP (2012). CDK2 and PKA mediated-sequential phosphorylation is critical for p19INK4d function in the DNA damage response. PLoS ONE.

[308] Bai F, Chan HL, Smith MD, Kiyokawa H, Pei XH (2014). *p19 *^*Ink4d*^ is a tumor suppressor and controls pituitary anterior lobe cell proliferation. Mol Cell Biol.

[309] Felisiak-Golabek A, Dansonka-Mieszkowska A, Rzepecka IK, Szafron L, Kwiatkowska E, Konopka B (2013). p19 ^INK4d^ mRNA and protein expression as new prognostic factors in ovarian cancer patients. Cancer Biol Ther.

[310] Morishita A (2011). Frequent loss of p19INK4D expression in hepatocellular carcinoma: relationship to tumor differentiation and patient survival. Oncol Rep.

[311] Parati MC, Pedersini R, Perego G, Reduzzi R, Savio T, Cabiddu M (2022). Ribociclib in the treatment of hormone-receptor positive/HER2-negative advanced and early breast cancer: overview of clinical data and patients selection. Breast Cancer Targets Therapy.

[312] Raheem F, Ofori H, Simpson L, Shah V (2022). Abemaciclib: the first FDA-approved CDK4/6 inhibitor for the adjuvant treatment of HR+ HER2−early breast cancer. Ann Pharmacother.

[313] Beaver JA, Amiri-Kordestani L, Charlab R, Chen W, Palmby T, Tilley A (2015). FDA approval: palbociclib for the treatment of postmenopausal patients with estrogen receptor-positive, HER2-negative metastatic breast cancer. Clin Cancer Res.

[314] Dhillon S (2021). Trilaciclib: first approval. Drugs.

[315] Mughal MJ, Bhadresha K, Kwok HF (2023). CDK inhibitors from past to present: a new wave of cancer therapy. Semin Cancer Biol.

[316] Schoninger SF, Blain SW (2020). The ongoing search for biomarkers of CDK4/6 inhibitor responsiveness in breast cancer. Mol Cancer Ther.

[317] Xu XQ, Pan XH, Wang TT, Wang J, Yang B, He QJ (2021). Intrinsic and acquired resistance to CDK4/6 inhibitors and potential overcoming strategies. Acta Pharmacol Sin.

[318] Papadimitriou MC, Pazaiti A, Iliakopoulos K, Markouli M, Michalaki V, Papadimitriou CA (2022). Resistance to CDK4/6 inhibition: Mechanisms and strategies to overcome a therapeutic problem in the treatment of hormone receptor-positive metastatic breast cancer. Biochimica et Biophysica Acta Mol Cell Res..

